# Gastrodin enhances stress resilience through promoting Wnt/β-Catenin-dependent neurogenesis

**DOI:** 10.1016/j.jare.2025.04.017

**Published:** 2025-04-13

**Authors:** Haili Zhang, Zhihuang Zhao, Pei Liu, Meidan Wang, Yu-e Liu, Hui He, Yangyan Ge, Tao Zhou, Chenghong Xiao, Zili You, Jinqiang Zhang

**Affiliations:** a Guizhou University of Traditional Chinese Medicine, Guiyang 550025, China; b The Center of Psychosomatic Medicine, Sichuan Provincial Center for Mental Health, Sichuan Provincial People’s Hospital, University of Electronic Science and Technology of China, Chengdu 6100544, China; c Faculty of Biology, University of Freiburg, Freiburg 79104, Germany

**Keywords:** Gastrodin, Stress resilience, Depression, Neurogenesis, Neural stem/precursor cells, Wnt/β-catenin

## Abstract

**Background:**

Enhancing stress resilience constitutes a pivotal strategy in mitigating the risk of depression, making it a critical component of both prevention and treatment. In the current work, we identified a compound, gastrodin (GAS), as capable of enhancing stress resilience, as demonstrated by its ability to protect against depression following chronic stress exposure.

**Objectives:**

To elucidate the potential of GAS to promote neurogenesis under chronic stress, along with the associated cellular and molecular processes involved.

**Method:**

We evaluated the effect of GAS on NSPC proliferation and differentiation using both *in vitro* and *in vivo* investigations. Neurogenesis was inhibited using temozolomide to verify GAS’s impact on stress resilience. Comprehensive methodologies, including hippocampal transcriptome analysis and western blotting, were utilized to identify the involvement of the Wnt/β-catenin pathway. Immunolocalization was conducted to confirm β-catenin’s nuclear translocation in SOX2^+^ cells within the hippocampal dentate gyrus subgranular zone.

**Results:**

GAS demonstrated robust stimulation of NSPC proliferation and neuronal differentiation, enhancing adult hippocampal neurogenesis under conditions of chronic stress. Inhibition of neurogenesis negated GAS’s protective effects on stress resilience. Integrated analysis pointed to the Wnt/β-catenin signaling pathway within NSPCs as a crucial mechanism facilitating GAS-promoted neurogenesis. Inhibiting Wnt expression or blocking β-catenin’s nuclear translocation abolished GAS’s neurogenic and stress-resilience enhancing effects.

**Conclusion:**

These results suggested that GAS directly activates the Wnt/β-catenin signaling pathway, which promotes the proliferation and neuronal differentiation of NSPCs, thereby enhancing adult hippocampal neurogenesis and promoting stress resilience to mitigate the risk of depression.

## Highlights

• Gastrodin enhances stress resilience in mice.
• Gastrodin protects adult hippocampal neurogenesis from chronic stress.
• Wnt/β-catenin-dependent neurogenesis mediates gastrodin’s effects.

## Graphical abstract

**Figure:**
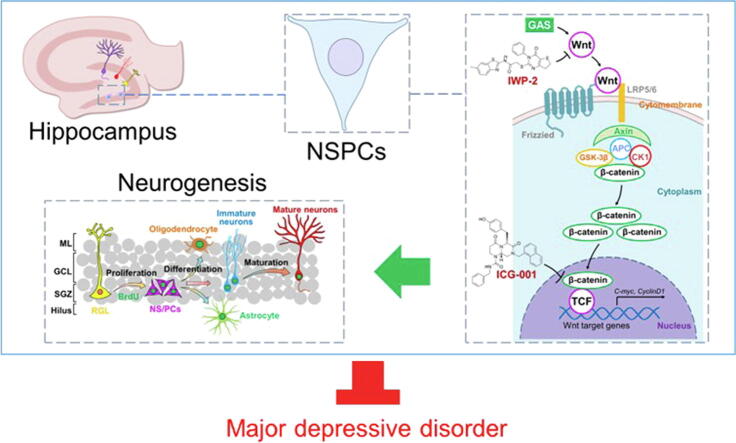

## Introduction

The major depressive disorder is a prevalent affective condition marked by profound and persistent depression, diminished interest, as well as cognitive and psychomotor retardation. In severe instances, it can result in functional impairment occupational impairment, and even contemplation of self-harm [1,2]. The World Health Organization (WHO) approximates that the global prevalence of depression reaches approximately 350 million individuals. Moreover, depression-related suicides claim over one million lives lost annually, resulting in an economic burden of up to one trillion US dollars per year [3].

The intricate etiology of depression which encompasses a multitude of physiological and psychological factors, constrains treatment options [4]. Therefore, elucidating the pathogenesis of depression and exploring novel molecular targets as well as more efficacious therapeutic drugs is imperative for the amelioration of depression. Research shows that most people with depression have experienced stressful events before the onset of depression [5,6]. For instance, the recent COVID-19 pandemic has imposed a substantial global burden of psychological distress. Between January 2020 and January 2021, a 27.6 % surge in the prevalence of major depression cases was observed worldwide [7]. Individuals react differently to the same stressors due to variances in personality traits, social experiences, genetic background, and other factors [8]. Some individuals initially display social avoidance and anxiety behaviors, which can eventually lead to the development of depression following a stressful event. These individuals demonstrate evident sensitivity to stress and are considered as part of the high-risk group for depression [9]. However, most individuals who encounter stress exhibit a rapid recovery from adverse experiences and demonstrate improved adaptability to stressful events, thereby developing resilience against chronic stress. Stress resilience refers to the capacity to effectively adapt when confronted with adversity, trauma, or significant threats [10,11]. Studies have found the inverse correlation between stress resilience and the risk of developing depression [12,13]. This highlights the paramount importance of strengthening individuals' stress resilience as a key strategy for effectively preventing depression, particularly within the current high-pressure social milieu.

Research has shown that adult hippocampal neurogenesis contributes to resilience to stress. Disrupting the internal environment of hippocampal neurogenesis through focal irradiation blocks neurogenesis, leading mice's stress resilience to decline and the emergence of depressive-like characteristics [14]. Most antidepressants promote neurogenesis in the adult hippocampus [[15], [16], [17]]. The therapeutic efficacy of antidepressants was nullified upon neurogenesis inhibition [18]. Enhancing hippocampal neurogenesis through environmental enrichment, physical exercise, or pharmacological intervention not only significantly augments stress resilience but also effectively ameliorates depression-like behaviors [[19], [20], [21]]. The findings suggest that neurogenesis is implicated, to some extent, in managing stress resilience and the development of depression. Consequently, the discovery and development of drugs that promote neurogenesis is an effective approach to enhancing individual stress resilience and reducing the risk of depression.

In recent years, herbal medicines and natural products have attracted much attention in promoting neurogenesis. These natural ingredients have become an important research direction for the treatment of nervous system diseases due to their diverse biological activities and relatively low side effects. Several natural compounds, such as curcumin, salidroside, and ginsenoside, have been shown to promote neurogenesis by modulating key signaling pathways, including Wnt/β-catenin, BDNF/TrkB, and PI3K/Akt [[22], [23], [24]]. These findings highlight the potential of natural products as promising strategies to promote neurogenesis and improve brain health. Gastrodin (GAS), an efficacious bioactive compound derived from the traditional Chinese medicine Gastrodiae Rhizoma, has shown a significant capacity to penetrate the blood–brain barrier (BBB) and exhibit therapeutic efficacy against neurological disorders [25,26].GAS is a promising TCM treatment because of its rapid onset of action, fewer side effects, and multi-target mechanisms to address the complex pathophysiology of depression compared with the current standard treatment for MDD [27]. Research indicates that GAS exerts neuroprotective effects by promoting hippocampal neurogenesis [[28], [29], [30]]. Despite the established beneficial effects of GAS on neuroprotection and promotion of hippocampal neurogenesis, the underlying mechanisms through which GAS promotes neurogenesis and its potential to elevate stress resilience remain unclear.

This research examines the cellular and molecular mechanisms underlying GAS-induced neurogenesis in both *in vitro* and *in vivo* settings, accessing its effects under pathophysiological and physiological conditions, as well as its impact on stress resilience. These findings will establish the groundwork for formulating intervention strategies to strengthen stress resilience and mitigate the risk of depression.

## Materials and methods

### Animals

Male C57BL/6J mice (8 weeks old) were purchased from Changsha Tianqin Biotechnology (Changsha, China). Mice were caged individually and resigned numbers for regrouping and recording the changes in the sucrose preferences, body weights and sucrose consumption before (1th week) and after stress (5th week). The mice were allowed to acclimate for at least seven days before the experiments' commencement. The mice were then habituated to a 1 % sucrose solution for 48 h with sucrose preference and body weight measurement weekly. Sucrose preference and body weight were recorded before the experiment began on day 0 and served as the baseline. All animal experiments were approved by the Institutional Animal Care and Use Committee of the Guizhou University of Traditional Chinese Medicine (Guiyang, Guizhou, China, No.20220137).

### Pharmacological treatments *in vivo*

#### Treatment with GAS in non-stress-exposed mice

Gastrodin (98 % pure) was obtained from Chengdu Alfa Biotechnology (AB0476, Chengdu, China). The mice were divided into five groups at random, exhibiting no discernible variations in body weight: treatment with saline (Control), a saline treatment group (Control), a group receiving 25 mg/kg of GAS (GAS/25), a group treated with 50 mg/kg of GAS (GAS/50), a group administered 100 mg/kg of GAS (GAS/100), and a group treated with 200 mg/kg of GAS (GAS/200). They received intraperitoneally administered saline, GAS (25, 50, 100 or 200  mg/kg/day), once daily for 5 weeks. To examine how GAS influences neurogenesis in typical physiological circumstances, they received intraperitoneally administered 5′-bromo-2′deoxyuridine (BrdU, B5002, Sigma-Aldrich, Missouri, USA) at a dosage of 50 mg/kg/d for seven consecutive days prior to sacrifice, following our previously published research [31]. During the initial week, five mice from each group were selected to analyze the proliferation of NSPCs. In the subsequent week, samples were chosen using the same method to evaluate the differentiation of NSPCs. In the fifth week, another set of five mice was selected for a focused examination of the maturity and survival of NSPCs.

#### Treatment with GAS and imipramine in stress-exposed mice

Gastrodin (98 % pure) was obtained from Chengdu Alfa Biotechnology (AB0476). Following a week of adaptive feeding, the mice were assigned randomly to seven group: non-stress + saline (Control), stress + saline (Stress), stress + 25  mg/kg of GAS (GAS/25), stress + 50  mg/kg of GAS (GAS/50), stress + 100  mg/kg of GAS (GAS/100), stress + 200  mg/kg of GAS (GAS/200), and stress + imipramine (IMI). They received intraperitoneal administered saline solution, GAS (25, 50, 100 or 200  mg/kg/day), or imipramine (10  mg/kg/day; Sigma-Aldrich, St. Louis, Missouri, USA) daily [32] at 17:00 h r a duration of 1 week. After that, the mice endured chronic stress for 4 weeks, as detailed in ***sections 2.3***. During chronic stress exposure, mice in each group continued to receive GAS or imipramine (I0971, TCI, Shanghai, China) treatment. Imipramine was used as a positive control in this study due to its well-documented ability to promote neurogenesis and enhance stress resilience [33,34]. This provides a reliable benchmark for the role of GAS in our behavioral and neurogenic assays. To investigate the effect of GAS on neurogenesis in a chronic stress exposure setting, BrdU (50 mg/kg/day) was injected intraperitoneally for 7 consecutive days before execution.

#### Treatment with temozolomide in GAS-treated mice

The possible influence of neurogenesis on improving stress resilience was examined through the use of the neurogenesis inhibitor temozolomide (TMZ, HY-17364, MCE, New Jersey, USA). The TMZ was dissolved in a 0.9 % saline solution supplemented with 5 % dimethyl sulfoxide (DMSO) at a final 2.5 μg/mL dosage. Following a week of adaptive feeding, the groups were designated as follows: non-stress + saline + DMSO (Control), stress + saline + DMSO (Stress), stress + GAS + DMSO (Stress + GAS), and stress + GAS + TMZ (Stress + GAS + TMZ). During the first week, mice in the Control and Stress groups received intraperitoneal injections of DMSO (at 10:00 h) and saline (at 16:00 h). Subsequently, continuous treatment with DMSO and saline was administered for a duration of 4 weeks. The Stress group underwent chronic stress for 4 weeks as described in ***sections 2.3***, while the Control group remained unexposed to any stress conditions. In the Stress + GAS group, an intraperitoneal injection of DMSO (at 10:00 h) was given in the morning followed by GAS administration (100 mg/kg/day) (at 17:00 h) during the initial week. For the subsequent four weeks, mice were subjected to chronic stress as outlined in ***sections 2.3*** while concurrently receiving GAS treatment. In addition, In the Stress + GAS + TMZ group, TMZ (25 μg/kg/day) was intraperitoneally injected at 10:00 h followed by GAS administration (100 mg/kg/day) at 17:00 h during the first week [35]. Four weeks later, under combination treatment of TMZ and GAS as described in ***sections 2.3***, the mice were exposed to chronic stress challenges. To assess the effect of TMZ blockade of neurogenesis on the proliferation and differentiation of mouse hippocampal neural stem cells, all mice were intraperitoneally injected with BrdU50 mg/kg/ day during the last week before sacrifice.

#### Treatment with IWP-2 or ICG-001

To study how Wnt/β-catenin signaling promotes neurogenesis and enhances stress resilience, we utilized IWP-2 (HY-13912, MCE), an inhibitor of Wnt, and ICG-001 (HY-14428, MCE), a β-catenin inhibitor. IWP-2 or ICG-001 was suspended in a solution of 0.9 % saline with 5 % DMSO at doses of 1  mg/mL and 0.5 mg/mL, individually. Following a week of adaptive feeding, the groups were designated as follows: non-stress + saline + DMSO (Control), stress + saline + DMSO (Stress), stress + GAS + DMSO (stress + GAS), stress + GAS + IWP-2 (stress + GAS + IWP-2), and stress + GAS + ICG-001 (Stress + GAS + ICG-001). The Control, Stress, and Stress + GAS groups were treated in accordance with the procedures described in ***sections 2.2.3***. In the Stress + GAS + IWP-2 group, intraperitoneal injections of IWP-2 (10 mg/kg/day) were administered at 10:00 h during the first week. Similarly, in the Stress + GAS + ICG-001 group, injections of ICG-001 (5 mg/kg/day) were given at 10:00 h during the first week [36,37]. Both groups were also administered intraperitoneal injections of GAS (100 mg/kg/day) at 17:00 h. During the subsequent four weeks, mice underwent chronic stress as outlined in ***sections 2.3*** while continuing with the aforementioned GAS or/and ICG-001 or IWP-2 treatment. After the mice were subjected to chronic stress for four weeks, BrdU administration was performed similarly as described above. One week before sacrifice, all mice received BrdU (50 mg/kg/ day, i.p.) to ensure the labeling of proliferating NSPCs for the duration of the experiment.

### Chronic stress

A chronic stress procedure was performed as previously described [38]. In brief, animals were exposed to two to three following stressors in random order: empty water bottles (12 h), food deprivation (12 h), tail clipping (10 min), restraint (2 h), lights- off for 3  h during the daylight phase, cage shaking (1 h), cage tilting (45°, 24 h), reversal of the light–dark cycle (24 h), strobe lighting (12 h), damp bedding (24 h), and a soiled cage (24 h), each period for 28 d.

### Behavioral tests

#### Open field test (OFT)

The open field test was used when following the previously outlined methodology [39]. The test chamber consisted of a 50 × 50 × 30 cm organic plastic box, equipped with an overhead camera to monitor the mice's movements. The mice were situated in the middle of the open field chamber and given a five-minute acclimatization period to the testing surroundings. For five more minutes, their exploratory activity was observed. The Autonomous Activity Monitor (Taimeng, Chengdu, China) was utilized to analyze various parameters including movement and rest time, dwell times in the center, the edges, and corners, the number of entries into the central region, total distance traveled, and average movement speed as evaluation metrics.

#### Elevated plus-maze test (EPMT)

The Elevated plus-maze test was replicated following the protocol outlined previously [40]. Two 35 × 5 cm open arms and two 35 × 5 cm enclosed arms connected by a 5 × 5-centimeter central zone made up the arrangement, which was 50 cm off the ground. Testing took place in a calm, softly illuminated setting. Prior to each trial, 75 % ethanol was used to sanitize the equipment. For five minutes, the mice's impromptu movements were monitored as they were carefully positioned in the central region, oriented toward one of the open arms. The admissions into the open arms and the total duration spent in that area were recorded using EMP100 software (Taimeng Tech, Chengdu, China).

#### Forced swimming test (FST)

The Forced swimming test was followed by the established protocol [41]. Each mouse was kept singly in a glass cylinder (25 cm in height and 15 cm in diameter) filled with water to a depth of 15 cm and kept at 26 °C for 10 min twenty-four hours before the experiment began. The experiment was carried out once more the next day for six minutes under the same setup. The duration of immobility, which encompasses both remaining still while floating and the movements necessary to maintain buoyancy, was recorded using YHFST software (Yihong Technology Co., Ltd., Wuhan, China) during the final 4 min of the swimming duration.

#### Sucrose preference test (SPT)

The sucrose preference test was performed as described [42]. Briefly, the mice were individually caged and subjected to food deprivation for 6 h and water for 12 h before the test. They were provided with access to water (A) and a 1 % sucrose solution (B) for 12 h. The sucrose preference was measured multiple times in the same animals, changing the location of the water and the sucrose solution once a week. The intake (g) of water and sucrose solution were recorded individually. To determine sucrose consumption, the body weight was measured between 14:00 and 15:00 on Monday weekly throughout the whole experiment. The sucrose preference was calculated each week for each mouse using the formula: 100 × [VolA/ (VolA + VolB)]. The sucrose consumption was normalized to body weight for each mouse.

#### Body weight and coat score

Coat score assay was performed as previous study described [43]. Mice were weighed weekly and physical appearance was evaluated in terms of the coat score. The total coat score was calculated as the sum of individual scores for the head, neck, forepaws, dorsal coat, ventral coat, hind paws, and tail. Animals were also assigned a “coat score” of 0 if they were unkempt or 1 if they were well-groomed.

#### Animal screening

Mice were treated with chronic stress for 4 weeks and then subdivided as described [44] into groups of SS or SR animals. The mice were considered SS if their immobility time in the FST and EPMT residence time in open-arm and their sucrose intake were within 1 SD of the mean values for control mice. If these values fell outside this threshold, then animals were considered SR.

### Cell culture and treatments

#### Neural stem/precursor cells (NSPCs) culture

NSPCs were isolated and cultivated from young adolescent C57BL/6J mice utilizing a previously established method [45] with minor adjustments. In summary, the complete subgranular zone (SVZ) was separated from brains that were cut sagittally in ice-cold DMEM/F12 (12400–024, Gibco, California, USA), minced into smaller pieces, and then subjected to digestion using 0.25 % pancreatin. Pipetting was then used to separate the minced tissue into individual cells. B27 (17504–044, Gibco) (×200), N2 (17502048, Gibco) (×100), 20 ng/ml FGF2 (450–33-50UG, Gibco), and 20 ng/ml EGF (315–09-500UG, Gibco) were added to high-glucose DMEM/F12 supplement, and the cells were cultivated for seven days at 37℃ with 5 % CO_2_. Following incubation, neurospheres were harvested through centrifugation (600 g) and 0.25 % pancreatin was used to enzymatically break down them into a single-cell suspension. The cells were then plated at a density of 5 × 10^4^ cells/cm^2^ in the proliferative medium. To facilitate serial passaging of the cells, the pancreatin dissociation procedure was performed three to four days.

#### Nspcs proliferation assay

After centrifuging at 500 rpm for 3 min, the supernatant was removed from the well-grown second-generation neuro progenitor cell aggregates. Subsequently, the cell pellet was then gently resuspended in 1 mL of trypsin (Gibco) and homogenized using a pipette. Following 5 min of enzymatic digestion, the reaction was stopped by introducing 3 mL of DMEM/F12 culture medium. The suspension was subjected to another centrifugation at 1000 rpm for a duration of five minutes, after which the supernatant was removed. Resuspend the cell pellet in 6 mL of cell proliferation culture medium and evenly distribute it into the wells of a 24-well plate. Cells were cultured under controlled conditions at 37℃ with 5 % CO_2_. Based on previous studies on the pharmacological activity of GAS in this range and preliminary *in vitro* experiments to confirm its efficacy and safety [46], we divided the experiments into four groups: three treatment groups with different doses of GAS (10, 50, and 100 μM) and three replicate wells each, as well as a control group that was cultivated in serum-free media. After 24 h, the diameter and quantity of neurospheres in each group were observed and recorded using a microscope to assess their proliferative activity.

#### Nspcs differentiation assay

Selected the second generation of vigorously proliferating neural stem cell spheres and discarded the supernatant after centrifugation at a speed of 500 rpm for three minutes. The cell pellet was suspended by gentle pipetting in a differentiation medium, composed of 93 % DMEM/F12, 5 % FBS, 1 % N-2, and 2 % B-27. Finally, continue centrifugation at a speed of 1000 rpm for five minutes to remove any residual liquid. The pellet obtained was re-suspended in the differentiation medium and then exposed to separate treatments using GAS concentrations of 10 μM, 50 μM, and 100 μM. Following the treatments, the cells were placed into a 24-well plate and incubated at 37℃ with 5 % CO_2_ to facilitate cellular differentiation. After 7 days, the culture medium should be removed and NSPCs fixed in 4 % paraformaldehyde for 30 min. The cells should then be rinsed three times with sterile PBS and permeabilized using a prepared solution of 0.5 % Triton X-100 for 15 min. Following another three PBS washes, the cells were treated with 10 % donkey serum for one hour to block. Following the removal of the blocking solution, the cells were incubated for an entire night at 4 °C with primary antibodies (rabbit anti-DCX, Catalog: 4604 s, Cell Signaling Technology, Massachusetts, USA; rabbit anti-NG2, Catalog: ab129051, Abacm, Massachusetts, USA; rabbit anti-GFAP, Catalog: 80788S, Cell Signaling Technology). The following day, the cells underwent three washes with PBS, each lasting five minutes, prior to incubation with secondary antibodies (DyLight 488-conjugate donkey anti-rabbit, Catalog: ab150073, Abcam) at room temperature in a light-free environment for ninety minutes. Finally, DAPI (Catalog:C1006, Beyotime, Shanghai, China) was applied for eight to fifteen minutes followed by washing thrice using sterile PBS before mounting slides for observation under an inverted fluorescence microscope.

### Western blotting

The mice were sedated with a 10 % solution of pentobarbital and then underwent transcardial perfusion with 0.9 % saline to cleanse the blood vessels. Following perfusion, the hippocampi were extracted and homogenized. The total protein of the hippocampal and NSPCs were extracted with RIPA lysate (Catalog: P0013B, Beyotime), and quantified by BCA quantification kit (Catalog: AR1189, Boster California, USA). Loading buffer (Catalog: 240003003, Solarbio, Beijing, China) was utilized to standardize the protein concentration and denature it. The proteins were then moved to PVDF membranes after being put onto SDS-PAGE (Catalog: 0000292059, Millipore, Massachusetts, USA). The membranes were incubated with skim milk for 30 min before being rinsed with Tris-buffered saline containing 0.1 % Tween-20 (TBST). The primary antibodies against Wnt (1: 1000, Catalog: ab219412, Servicebio, Wuhan, China), β-catenin (1: 2000, Catalog: GB12015-100, Servicebio), p-β-catenin (1: 1000, Catalog: PA5-77933 Servicebio), C-Myc (1: 1000, Catalog: GB113748-100, Servicebio), and Cyclin D1 (1: 1000, Catalog: GB113748-100, Servicebio) were then incubated with blots overnight at 4℃. The membranes underwent three TBST washes before being incubated for an additional hour with a secondary antibody (1:10,000; Catalog: GB23303, Servicebio) for an additional 1 h. Finally, enhanced chemiluminescence (Catalog: BMU102-CN, Abbkine, Wuhan, China) was used to develop the membranes for 1–2 min. The ChemiDoc Touch system from Bio-Rad was used to visualize the blots, and the band intensity was visualized using Alpha software (version 1.45 J; National Institutes of Health, Bethesda, MD, USA).

### Immunohistochemistry

The groups of mice (5 animals each) underwent deep anesthesia with 10 % pentobarbital, given at a dosage of 10 ml/kg, before being perfused for 6 min with phosphate-buffered saline (PBS; pH 7.2). Subsequently, they were perfused with 4 % paraformaldehyde (PFA; pH 7.2) for an additional 6 min. After being removed, the brains were preserved for 72 h in 4 % PFA and then dehydrated for another 72 h in a 30 % sucrose solution. Prior to use, the brains underwent swift freezing and were preserved at a temperature of −80°C. To generate coronal cryostat sections, which had a thickness of 25 μm each, we employed a sliding vibratome (CM1900; Leica Microsystems, Wetzlar, Germany). The resulting sections were placed in a 12-well plate that had six consecutive slices and was maintained at 4 °C. The plate was filled with PBS that contained 0.02 % sodium azide.

Every sixth section of the brain (25 μm thick) that contains the hippocampus were selected for immunostaining to measure the overall cell populations within this region (BrdU^+^-SOX^+^ cells, BrdU^+^-GFAP^+^ cells, BrdU^+^-DCX^+^ cells, BrdU^+^-Olig2^+^ cells, BrdU^+^-NeuN^+^ cells, BrdU^+^ cells). Hippocampus slices were exposed to 0.5 % Triton X-100 in PBS for 15 min. This was succeeded by a blocking step using 10 % donkey serum (Solarbio) for one hour before being incubated for an entire night at 4 °C (both 1:400; rabbit anti-SOX2, Catalog: OB-PRB110-01, Biofarm, Hangzhou, China; rabbit anti-GFAP, Catalog: 80788S, Cell Signaling Technology; rabbit anti-DCX, Catalog: 4604 s, Cell Signaling Technology; rabbit anti-Olig2, Cell Signaling Technology; rabbit anti-NeuN, Catalog: 24307 s, Cell Signaling Technology; rat anti-BrdU, Abcam). The next day, the slices were subjected to treatment with either the DyLight 549- or 488-conjugated secondary antibody (all at a dilution of 1:300, Jackson ImmunoResearch, USA) for 2 h avoiding the light. Following a 5-minute staining process with 4′, 6-diamidino-2-phenylindole (DAPI; Beyotime), cells were analyzed using a fluorescent microscope (Olympus IX 73).

### Transcriptome sequencing and analysis

Hippocampal sampling and animal perfusion were performed as outlined in the reference [31]. Total RNA extraction was performed using Trizol (Thermo Fisher, MA, USA), while the integrity of the RNA and possible contamination were evaluated with the Bioanalyzer 2100 (Agilent, CA, USA). The NanoDrop ND-1000 (NanoDrop, DE, USA) was used to measure the concentration and purity of RNA. Later, Shanghai, China-based Majorbio Bio-Pharm Technology Co., Ltd. used the Illumina NovaSeq™ 6000 platform to sequence the RNA libraries. The NCBI Sequence Read Archive has received the raw sequencing data (BioProject ID PRJNA1174085).

Enrichment analysis for GO and KEGG with respect to the targets was performed using the cluster profile R package, which includes the three facets of GO analysis: biological processes (BP), molecular functions (MF), and cellular components (CC). The results were displayed using the “ggplot2″ R package after filtering them at a significance level of P < 0.05. We employed Fisher's Exact Test to evaluate whether a KEGG pathway exhibits a higher degree of enrichment in a set of differentially expressed genes compared to what would be expected by chance. To account for the possibility of false discoveries, we utilized the Benjamini-Hochberg (BH) approach to modify the P-values in light of multiple comparisons. A KEGG pathway is considered highly enriched in differentially expressed genes if its adjusted P-value (adj.P) is 0.05 or lower, suggesting its relevance to the biological conditions or states being studied.

### Molecular docking and dynamics of GAS binding to Wnt

Opening the 3D structure of the Wnt protein (PDB ID: 6AHY) in PyMOL 2.6 for subsequent preprocessing, which involves removing water molecules and impurities, eliminating unnecessary chains, filling in missing residues, and adding hydrogen atoms. Docking preparation was performed using AutoDockTools-1.5.7 by importing the preprocessed Wnt protein and GAS, calculating Gasteiger charges, and exporting them in PDBQT format. A grid box was defined to cover the active sites of the Wnt protein, ensuring comprehensive sampling of potential binding regions. Docking parameters were meticulously set prior to conducting molecular docking with AutoDock, which generated multiple docking poses along with their corresponding energy scores. The most favorable docking pose was visualized using PyMOL 2.6, and a detailed two-dimensional interaction map was subsequently generated with LigPlot + v.2.2 to systematically analyze hydrogen bonds and hydrophobic interactions.

Molecular dynamics simulations were performed using GROMACS 2022.3. The GAFF force field parameters and RESP charges for the GAS ligand were optimized using AmberTools 22 and Gaussian 16 W, and subsequently incorporated into the topology file. All simulations were carried out under conditions of 300 K and 1 bar. The Amber99sb-ildn force field was employed for the Wnt protein, while the TIP3P model was used for water molecules. Sodium ions (Na^+^) were added to neutralize the system charge. After energy minimization, equilibration was conducted in two stages: 100,000 steps of NVT followed by 100,000 steps of NPT. A production simulation lasting 500 ns was then performed. Finally, trajectory analysis and root-mean-square deviation (RMSD) calculations were carried out. Ligand binding stability was evaluated using the MM/PBSA method.

### Statistical analyses

The quantitative analysis of positive staining, cell counts, and relative fluorescence intensity was conducted using Image J (version 1.45 J; National Institutes of Health, Bethesda, MD, USA) along with Image-Pro Plus 6.0 (Media Cybernetics, Rockville, MD, USA), as outlined in earlier studies [47].

Quantitative outcomes were expressed as means ± standard error of the mean (SEM). The statistical analyses were performed with the use of GraphPad Prism software (version 8.0, GraphPad Software Inc, Chicago, IL, USA). One-way or two-way ANOVA, accompanied by Tukey’s post hoc tests, was employed to evaluate comparisons among three or more values. To assess significance in pairwise comparisons, the Student’s two-tailed *t*-test was applied, establishing a threshold of P < 0.05 set for determining statistical significance.

## Results

### Gastrodin promoted the proliferation of NSPCs and neurogenesis *in vitro* and *in vivo*

**Fig. 1:**
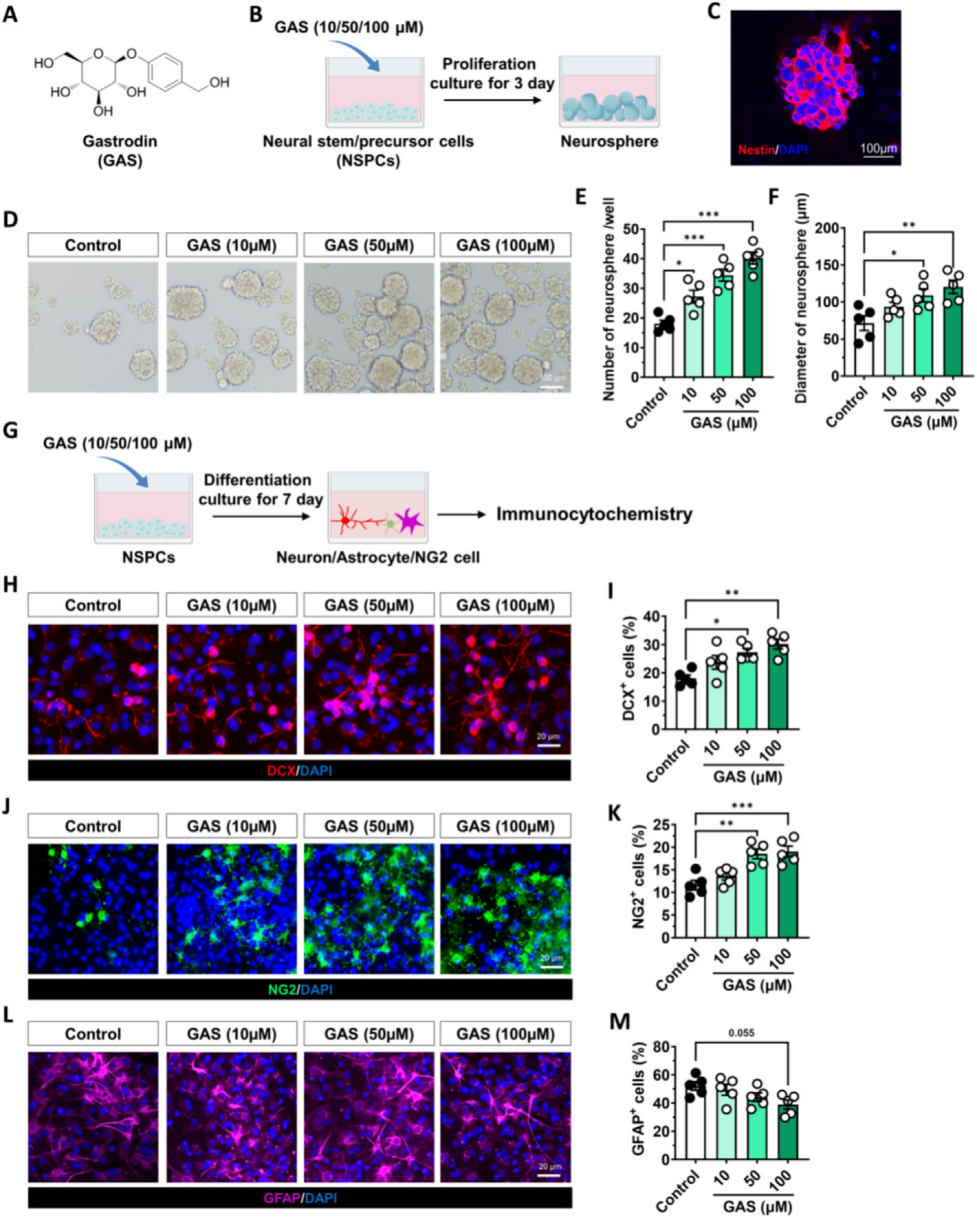
**Gastrodin promoted the proliferation of NSPCs and neurogenesis *in vitro* (A)** The structure of Gastrodin (GAS). **(B)** Scheme for evaluating the effects of GAS on neural stem/precursor cells (NSPCs) proliferation. **(C)** The identification of NSPCs is carried out using Nestin (red) labeling. **(D-F)** Effects of GAS on the number and diameter of neurosphere. **(G)** Scheme for evaluating the effects of GAS on NSPC differentiation. **(H-M)** Effects of GAS on the differentiation of NSPCs into neurons (DCX-positive cells, red), oligodendrocytes (NG2-positive cells, green), and astrocytes (GFAP-positive cells, pink). Data are mean ± standard error of the mean (SEM). *p < 0.05, ^**^p < 0.01, ^***^p < 0.001 vs Control microglia conditioned medium (PBS-M−CM), (one-way ANOVA with Tukey’s multiple-comparisons test). Details of the statistical analyses are provided in Supplementary Table 1. (For interpretation of the references to colour in this figure legend, the reader is referred to the web version of this article.)

We investigated the impact of GAS on the proliferation and differentiation of neural stem/precursor cells (NSPCs) *in vitro* and *in vivo* in order to determine the regulatory role of GAS on NSPCs. To assess whether GAS promotes NSPC proliferation, we cultured NSPCs in a medium with or without GAS for 3 days **(**Fig. 1**A–C)**. Under *in vitro* culture conditions, treatment with 10, 50, or 100 μM GAS significantly increased the number of neurospheres. Moreover, exposure to either 50 or 100 μM of GAS effectively enhanced the diameter of neurospheres **(**Fig. 1**D–F)**. To confirm the potential of GAS in promoting NSPC differentiation, NSPCs were cultivated in a differentiation medium with or without GAS for 7 days **(**Fig. 1**G)**. The proportions of cells that were committed to developing into neurons (DCX^+^ cells), NG2 glial cells (NG2^+^ cells), or astrocytes (GFAP^+^ cells). Treatment with 50 or 100 μM of GAS significantly promoted NSPCs differentiation into neuron and NG2 glia **(**Fig. 1**H–M)**. These findings indicate that GAS exerts an effect on the regulation of NSPCs' function, including through promoting their proliferation and differentiation.

**Fig. 2:**
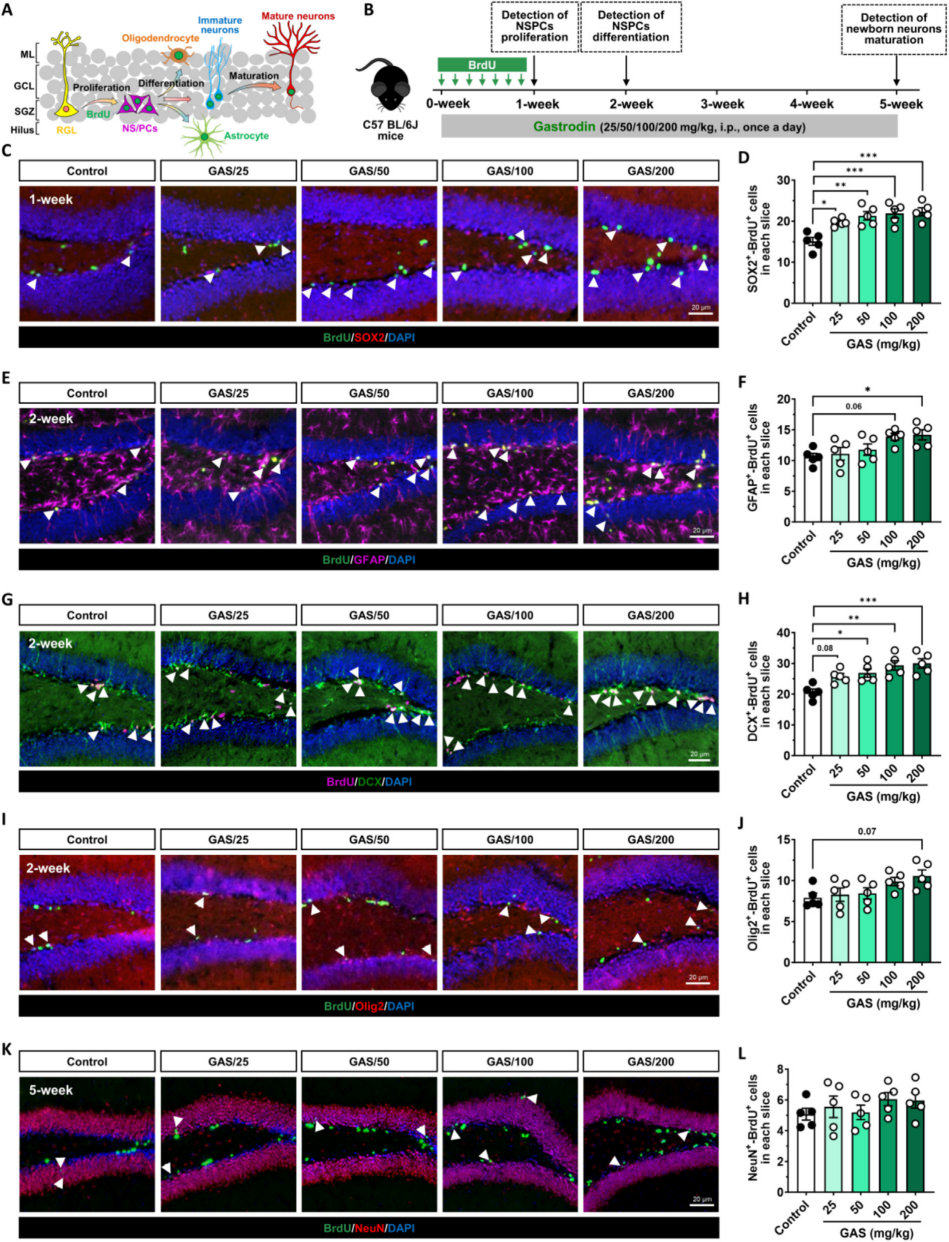
**Gastrodin promoted the proliferation of NSPCs and neurogenesis *in vivo* (A)** Schematic diagram of the fate of proliferating neural stem/precursor cells (NSPCs) traced with 5′- bromo- 2′deoxyuridine (BrdU). **(B)** Scheme for exploring the effect of Gastrodin (GAS) on NSPCs proliferation, differentiation, and newborn neuron maturation in the hippocampus DG of mice. **(C-D)** Effects of GAS on the proliferation of NSPCs in hippocampus of mice. The proliferating NSPCs were labeled both with SOX2 and BrdU (white arrowheads suggest), and their number was quantified. **(E-J)** Effects of GAS on the differentiation of NSPCs in hippocampus of mice. The astrocytes differentiated from NSPCs were labeled both with GFAP and BrdU, newborn immature neurons differentiated from NSPCs were labeled both with DCX and BrdU, the oligodendrocyte differentiated from NSPCs were labeled both with Olig2 and BrdU, and their number was quantified. **(K-L)** Effects of GAS on the newborn neuron maturation of NSPCs in hippocampus of mice. The newborn mature neurons were labeled with both BrdU and NeuN (White arrowheads suggest), and their number was quantified. Data are mean ± standard error of the mean (SEM). ns, not significant, *p < 0.05, ^**^p < 0.01, ^***^p < 0.001 vs Control group (one-way ANOVA with Tukey's multiple-comparisons test). Details of the statistical analyses are provided in Supplementary Table 2.

Subsequently, 5-Bromo-2′-deoxyuridine (BrdU) was employed to track the destiny of proliferating NSPCs in the hippocampus, with the objective of assessing how GAS affects the proliferation, differentiation, and survival of NSPCs *in vivo* **(**Fig. 2**A–B)**. Immunofluorescence staining reveals that treatment with GAS significantly boosts the quantity of BrdU^+^-SOX^+^ cells (proliferating NSPCs) **(**Fig. 2**C, D)**, BrdU^+^-GFAP^+^ cells (astrocyte differentiated from proliferating NSPCs) **(**Fig. 2**E, F)** and BrdU^+^-DCX^+^ cells (newborn immature neurons differentiated from NSPCs) in hippocampus with the most significant effects with a GAS dosage of 200 mg/kg **(**Fig. 2**G, H)**. The number of BrdU^+^-Olig2^+^ cells (oligodendrocytes differentiated from NSPCs) increased after receiving 200 mg/kg of GAS, even though this change was not statistically significant (*p* < 0.05) **(**Fig. 2**I, J)**. Notably, no significant variation was detected in the quantity of BrdU^+^-NeuN^+^ cells (newborn mature neurons) within the hippocampus **(**Fig. 2**K, L)**, indicating that the maturation process of newborn immature neurons additionally increased by GAS was restricted under healthy physiological conditions. During adult hippocampal neurogenesis, 60 % to 80 % of new neurons are eliminated by apoptosis, ensuring only those successfully integrated into neural networks survive and mature [48].

These results imply that, in healthy physiological settings, GAS does not affect the maturation process of newborn immature neurons but rather stimulates the proliferation and neuronal differentiation of NSPCs.

### Gastrodin enhances stress resilience by promoting neurogenesis in the adult hippocampus

**Fig. 3:**
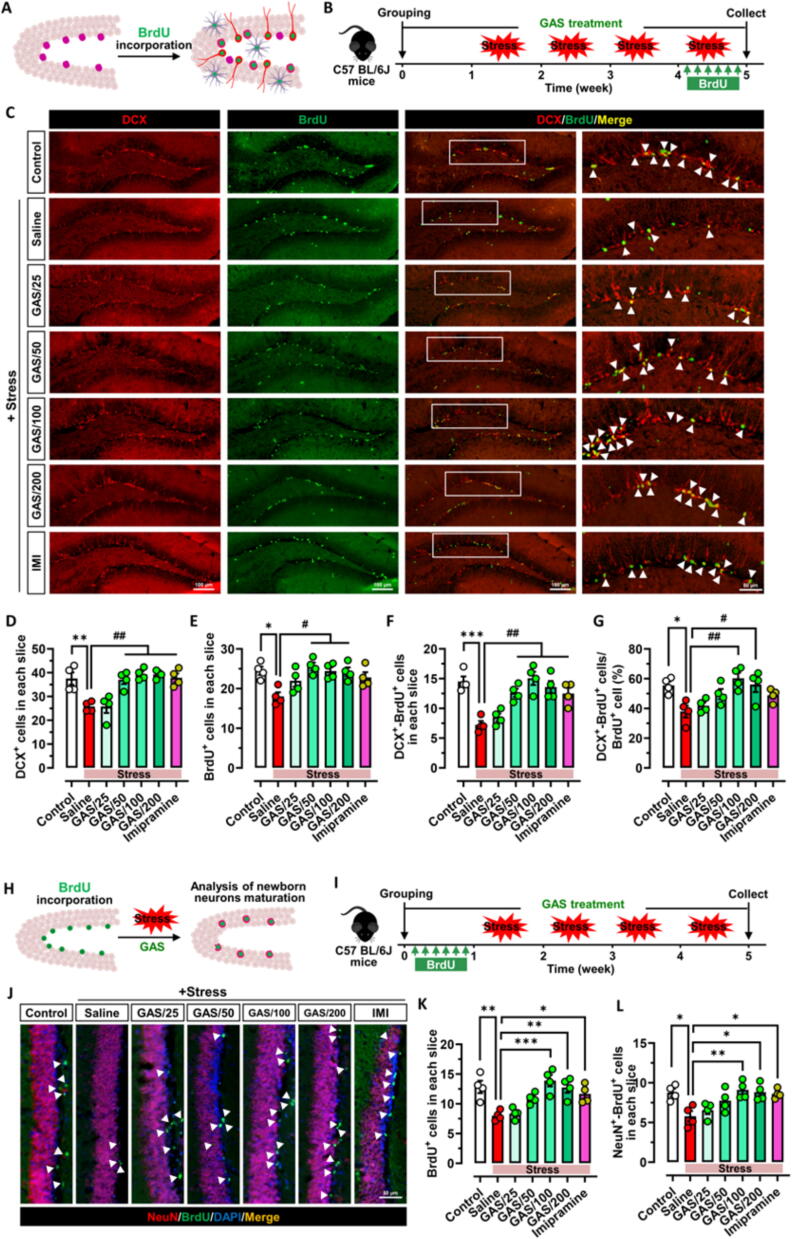
**Gastrodin protects adult hippocampal neurogenesis from chronic stress (A-B)** Scheme for exploring the effect of Gastrodin (GAS) on the adult hippocampal neurogenesis of stress-exposed mice through BrdU integration. **(C-G)** Effects of GAS on the proliferation and differentiation of NSPCs in hippocamous of stress-exposed mice. Proliferating NSPCs were labeled with BrdU (green), immature neurons were labeled with doublecortin (DCX, red), newborn immature neurons differentiated from NSPCs were labeled both with DCX and BrdU, and their number was quantified. **(H-I)** Scheme for exploring the effect of GAS on the maturation of newborn neurons in hippocampus of stress-exposed mice through BrdU integration. **(J-L)** Effects of GAS on the number of surviving proliferating cells (BrdU^+^ cells) and newborn mature neurons (BrdU^+^-NeuN^+^ cells) in hippocampus of stress-exposed mice. Data are mean ± standard error of the mean (SEM). ns, not significant, *p < 0.05, ^**^p < 0.01, ^***^p < 0.001 vs Control group, ^#^*p* < 0.05, ^##^*p* < 0.01 vs Stress group (**D** − **G**), *p < 0.05, ^**^p < 0.01, ^***^p < 0.001 vs Stress group (**K** and **L**: one-way ANOVA with Tukey's multiple-comparisons test). Details of the statistical analyses are provided in Supplementary Table 3. (For interpretation of the references to colour in this figure legend, the reader is referred to the web version of this article.)

Building on our earlier observations that GAS promotes the proliferation and neuronal differentiation of NSPCs under healthy physiological conditions, we sought to investigate the influence of GAS on hippocampus neurogenesis under chronic stress exposure **(**Fig. 3**A and B)**. The findings indicated that stress exposure notably diminished the quantity of DCX^+^ cells, BrdU^+^ cells and DCX^+^-BrdU^+^ cells within the hippocampus of stressed mice. However, even when chronic stress exposure was present, animals treated with GAS (50, 100, or 200 mg/kg) or IMI showed no loss in hippocampal size **(**Fig. 3**C–F)**. The chronic stress significantly suppressed the proportion of NSPC differentiation into neurons in the hippocampus's SGZ, indicating suppression of both NSPC proliferation and neuronal differentiation. Nonetheless, treatment with GAS at doses of 100 or 200 mg/kg prevented the reduction in the rate of neuronal differentiation in proliferating NSPCs within the hippocampus of stressed mice **(**Fig. 3**G)**.

Given that GAS did not affect the maturation process of newborn immature neurons under healthy physiological conditions, we additionally investigate how GAS influences the development of newly formed neurons when subjected to prolonged stress exposure. BrdU was injected into GAS-treated mice before stress exposure and these mice followed with 4 weeks of chronic stress exposure **(**Fig. 3**H–I).** The surviving proliferating cells (BrdU^+^ cells) and newborn mature neurons (BrdU^+^-NeuN^+^ cells) in hippocampus of each group mice were examined **(**Fig. 3**J)**. The findings indicated that chronic stress considerably diminished the quantity of surviving proliferating cells (BrdU^+^ cells) and newborn mature neurons (BrdU^+^-NeuN^+^ cells) in the hippocampal dentate gyrus. However, a decrease was not noted in the hippocampus mice receiving 100 or 200 mg/kg GAS or IMI, even under chronic stress exposure **(**Fig. 3**K–L)**.

These findings suggest that GAS promotes the NSPCs proliferation and neuronal differentiation in the hippocampal DG and the maturation and survival of newborn immature neurons under chronic stress exposure.

**Fig. 4:**
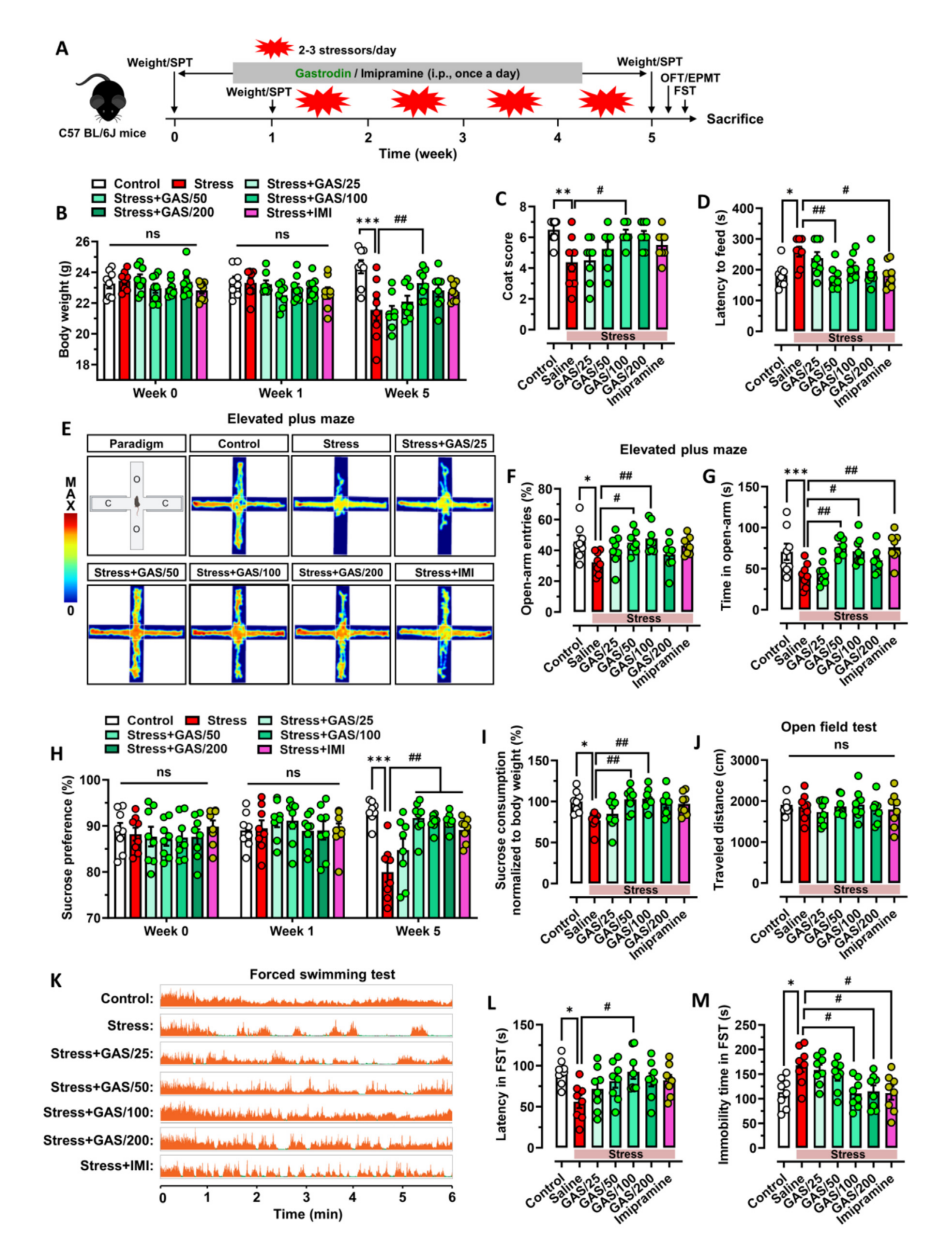
**Gastrodin elevates the resilience to stress in mice (A)** Scheme of the experimental procedure. The mice were either stressed or not in the absence or presence of Gastrodin (GAS). SPT, sucrose preference test; OFT, open field test; EPMT, elevated plus maze test; FST, forced swimming test. **(B)** Effects of GAS on the body weight of mice at baseline (0  week), prior to treatment (1  week), or following treatment (5  weeks). **(C-D)** Quantification of coat score of each group mice and latency to feed in novelty-suppressed feeding test. **(E-G)** Effects of GAS on anxiety-like behaviors of stress-exposed mice in the elevated plus maze test. On the left is the thermogram of movement trajectories of mice in each group in elevated plus maze test **(E)** On the right is the histogram showing the time in open-arm **(F)** and the change in open-arm entries **(G)** in the elevated plus maze test. **(H)** Effects of GAS on the the sucrose preference of mice at baseline (0  week), prior to treatment (1  week), or following treatment (5  weeks). **(I-J)** Quantification of sucrose consumption standardized to their body weight and the traveled distances in the open field. **(K-M)** Effects of GAS on depressive-like behaviors of stress-exposed mice in the forced swimming test. On the left is the dop diagram of the energy expenditure of mice during the forced swimming test **(K)**. On the right is the histogram showing the change in latency **(L)** and immobility time **(M)** of each group mice in the forced swimming test. Data are mean ± standard error of the mean (SEM) (n = 8 mice for each group). ns, not significant, *p < 0.05, ^**^p < 0.01, ^***^p < 0.001 vs Control group, ^#^*p* < 0.05, ^##^*p* < 0.01 vs Stress group (**B** and **H**: two-way ANOVA with Tukey's multiple-comparisons test; **C, D, F, G, I, J, L** and **M**: one-way ANOVA with Tukey's multiple-comparisons test). Details of the statistical analyses are provided in Supplementary Table 4.

Given that GAS promotes hippocampal neurogenesis, we next use a range of behavioral assessments to examine how GAS affects mice's ability to withstand stress-induced depression and anxiety. **(**Fig. 4**A)**. Body weight demonstrated no significant differences among the groups prior to intervention. One week of GAS subsequent administration did not exert a direct impact on the weight of mice. However, after four weeks of stress exposure, stressed mice exhibited a notable reduction in weight and coat score compared to the control group. Notably, this stress-induced weight loss and coat score reduction were prevented in mice protected by 100 mg/kg of GAS, while lower doses of GAS proved failed to fully mitigate the weight loss induced by chronic stress **(**Fig. 4**B–C)**. In the novel environment-suppressed feeding experiment, stress-exposed mice displayed an extended period of delayed feeding latency, a phenomenon significantly absent in GAS (50 mg/kg) or IMI treatments **(**Fig. 4**D)**.

Exposure to chronic stress has been demonstrated to decrease the duration of time spent and the number of entries into the open arms during anxiety-related behaviors in mice, as assessed by the elevated plus maze test **(**Fig. 4**E)**. About 50 % of the mice showed significant anxiety-like behavior **(Fig. S1A, B)**. As anticipated, these stress-induced anxiety-like behaviors were absent in GAS-treated mice (50 or 100 mg/kg) even under chronic stress exposure **(**Fig. 4**F, G)**. The sucrose preference test, utilized to evaluate anhedonia in mice in mice, showed no notable variations in sucrose preference between the groups before the GAS treatment. After one week of GAS administration, no apparent direct effects were observed on sucrose preference in mice. However, after four weeks of stress exposure, the stress-exposed mice showed a decreased sucrose preference in comparison to the control animals, and almost all of them showed marked symptoms of anhedonia **(Fig. S1C, D)**. Furthermore, we observed that the proportion of individuals exhibiting anhedonia was significantly reduced in mice treated with GAS (50, 100, or 200 mg/kg) or IMI, as evidenced by a markedly higher sucrose preference compared to stress-exposed controls **(**Fig. 4**H)** and similar trends were observed in sucrose consumption preference **(**Fig. 4**I)**. Conversely, neither GAS nor IMI had effects on the distance covered during the open field test **(**Fig. 4**J)**. These results indicated that GAS intervention did not show obvious side effects such as sedation or increased excitability in stress-exposed mice. Stress-exposed mice were subjected to an extended period of immobility and lower latency in the forced swimming test, which measures behavioral despair in these animals. More than 70 % of the mice showed significant depressive-like behavior **(Fig. S1E, F)**. As anticipated, these stress-induced depressive-like behaviors were notably missing in GAS (100 or 200 mg/kg) mice exposed to stress, while lower doses failed to provide sufficient protection against depressive-like behavior triggered by chronic stress **(**Fig. 4**K–M)**.

These results suggest that GAS, particularly at the dose of 100 mg/kg enhances the stress resilience of mice and protects them from stress-depression.

**Fig. 5:**
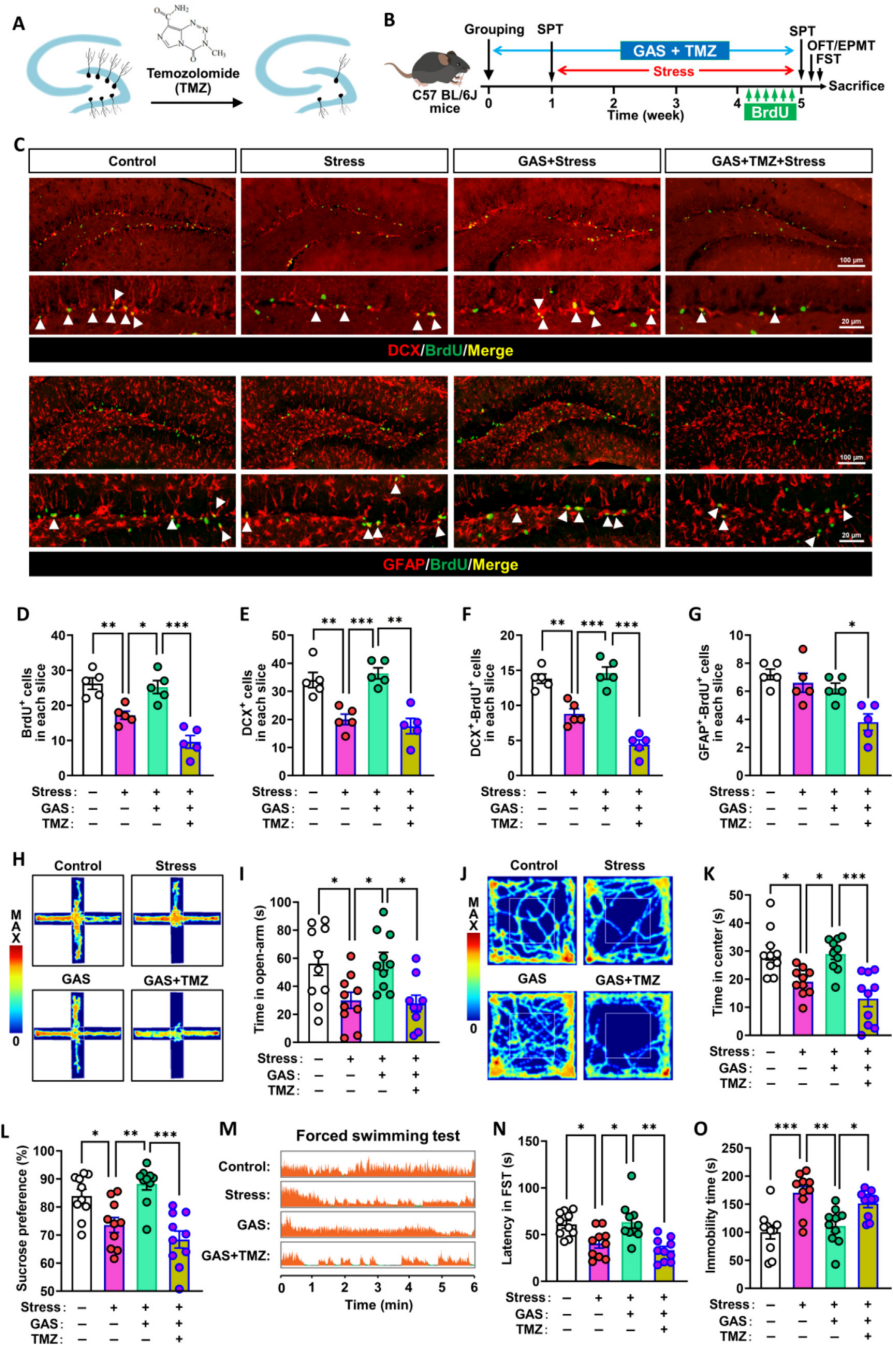
**The enhancement of stress resilience by gastrodin is partly dependent on neurogenesis in the adult hippocampus (A-B)** Scheme for blocking neurogenesis with Temozolomide (TMZ) in stress-exposed mice treated with Gastrodin (GAS). SPT, sucrose preference test; OFT, open field test; EPMT, elevated plus maze test; FST, forced swimming test. **(C-G)** Effects of TMZ treatment on the proliferation and differentiation of NSPCs in hippocampus of stress-exposed mice treated with GAS. Proliferating NSPCs were labeled with BrdU (green), and immature neurons were labeled with Doublecortin (DCX), astrocyte were labeled with glial fibrillary acidic protein (GFAP), the astrocytes differentiated from NSPCs were labeled both with GFAP and BrdU, newborn immature neurons differentiated from NSPCs were labeled both with DCX and BrdU, and their number was quantified. **(H-K)** Effects of blocking neurogenesis on anxiety-like behaviors of stress-exposed mice treated with GAS in the elevated plus maze test and open field test (n = 8). **(L)** Effects of blocking neurogenesis on the sucrose preference of stress-exposed mice treated with GAS. **(M−O)** Effects of blocking neurogenesis on behavioral despair in the forced swimming test of stress-exposed mice treated with GAS. Data are mean ± standard error of the mean (SEM). *p < 0.05, ^**^p < 0.01, ^***^p < 0.001 (one-way ANOVA with Tukey's multiple-comparisons test). Details of the statistical analyses are provided in Supplementary Table 5. (For interpretation of the references to colour in this figure legend, the reader is referred to the web version of this article.)

To investigate whether the enhancement of stress resilience by GAS depends on hippocampal neurogenesis, we employed Temozolomide (TMZ) to eliminate neurogenesis in stress-exposed mice and followed it with GAS **(**Fig. 5**A, B)**. In the hippocampal SGZ of mice treated with GAS, the findings demonstrated that TMZ administration significantly lowered the quantities of BrdU^+^, DCX^+^, DCX^+^-BrdU^+^ cells, and BrdU^+^-GFAP^+^ cells in addition to a decrease in the pace of neuronal development **(**Fig. 5 **C–G),** suggesting that TMZ successfully blocked the enhancing influence of GAS on neurogenesis in the adult hippocampus of stress-exposed mice. Significantly, inhibition of neurogenesis through TMZ negated the positive influence of GAS on stress resilience, as evidenced by a reduction in the duration spent in open arms during the elevated plus maze test time spent in the center during the open field test, sucrose preference, and prolonged periods of immobility during the forced swimming test when compared with GAS-treated mice **(**Fig. 5 **H–O)**.

The results suggest that the improvement of stress resilience through GAS is somewhat reliant on neurogenesis in the adult hippocampus.

### Wnt/β-catenin signaling is critical in gastrodin protect hippocampal neurogenesis from stress

**Fig. 6:**
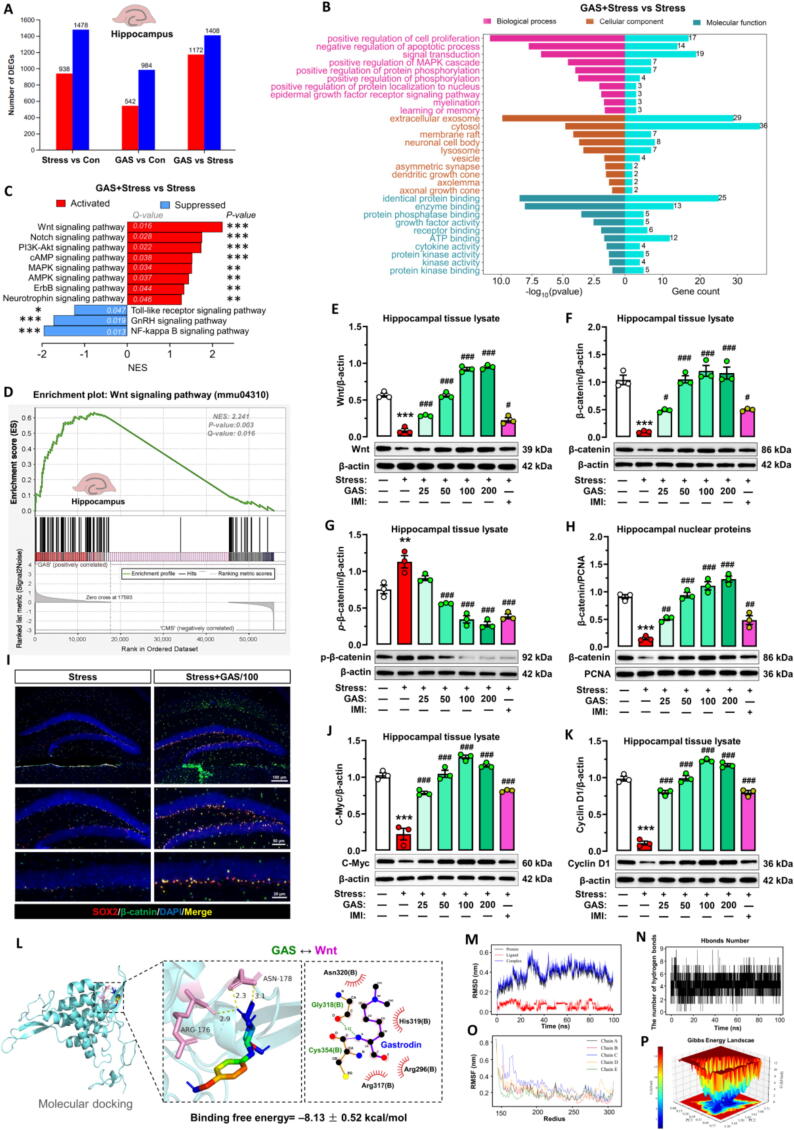
**Gastrodin activated the Wnt/β-catenin signaling pathway in hippocampus of stress-exposed mice (A)** Bar graph displaying the numbers of differentially expressed genes (DEGs) in hippocampus of control (Con), stress and Gastrodin + Stress (GAS). **(B-C)** GO and KEGG enrichment analyses of DEGs between the GAS + Stress and Stress groups. **(D)** Gene set enrichment analysis shows that the hippocampus transcriptomes of GAS mice and Stress animals have a more abundant Wnt signaling pathway. **(E-H, J and K)** Western blotting showing the changes in the levels of Wnt, β-catenin, *p*-β-catenin, C-Myc and Cyclin D1 in hippocampus of stress-exposed mice (n = 3, each sample in triplicate). Data are mean ± standard error of the mean (SEM). ^**^p < 0.01, ^***^p < 0.001 vs Control group, ^#^*p* < 0.05, ^##^*p* < 0.01, ^###^*p* < 0.001 vs Stress group (one-way ANOVA with Tukey's multiple-comparisons test). Details of the statistical analyses are provided in Supplementary Table 6. **(I)** Multiple immunofluorescence revealed β-catenin (green) expression in NSPCs (SOX^+^ cells) of the dentate gyrus in stress-exposed mice treated with GAS. **(L-P)** Molecular docking **(L)** and dynamics **(M−P)** of GAS binding to Wnt. (For interpretation of the references to colour in this figure legend, the reader is referred to the web version of this article.)

Through transcriptome sequencing, a comparison of RNA sequences from the hippocampus of Control, Stress, and Stress + GAS mice showed that 2416 genes were expressed differently in the Stress group compared to the Control group. Furthermore, 1516 differentially expressed genes were identified between the GAS + Stress and Stress groups in mice, including 542 upregulated and 984 downregulated genes **(**Fig. 6**A)**. The findings from the GO analysis indicated that the majority of differentially expressed genes between the GAS + Stress group and the Stress group were linked to the enhancement of cell proliferation and the suppression of apoptotic processes **(**Fig. 6**B)**. Employing multichannel gene set enrichment analysis (GSEA), our investigation revealed that transcripts found in the hippocampus of mice subjected to Stress + GAS showed a significant enrichment in genes associated with the activation of various signaling pathways, including Wnt, Notch, PI3K-Akt, cAMP, MAPK, ErbB and Neurotrophin signaling pathways while inhibiting Toll-like receptor, GnRH and NF-kappa B signaling pathways **(**Fig. 6**C)**. The Wnt signaling pathway exhibited the most significant upregulation and enrichment among the analyzed pathways **(**Fig. 6**D)**.

Considering the essential function of Wnt/β-catenin signaling in NSPCs induction, we examined whether GAS acts to protect hippocampal neurogenesis through this signaling. Western blot analysis revealed that chronic stress significantly decreased Wnt and β-catenin expression in hippocampal lysates, and nuclear β-catenin expression in hippocampal cells while increasing phosphorylated β-catenin (*p*-β-catenin) expression compared to control mice. Dramatically, GAS (50–200 mg/kg) significantly increased Wnt and β-catenin within the hippocampal lysates and boosted β-catenin levels in the nuclei of hippocampal cells, while reduced *p*-β-catenin expression in the hippocampal lysates compared to stress mice **(**Fig. 6**E–H)**. Immunolocalization results further confirmed that GAS significantly promoted the nuclear translocation of β-catenin in SOX2^+^ cells in the SGZ of hippocampal DG **(**Fig. 6**I)**. A significant rise in the expression levels of target genes located downstream, specifically C-Myc and Cyclin D1, was linked to this activation of the Wnt/β-catenin signaling pathway. **(**Fig. 6**J–K)**.

The results of the molecular docking studies revealed a stable interaction between GAS and Wnt, with a stability value of −8.13 ± 0.52 kcal/mo **(**Fig. 6**L)**. Furthermore, this study provided additional evidence supporting the strong binding affinity between GAS and the Wnt and β-catenin protein through molecular dynamics simulations **(**Fig. 6**M–P)**. This stable binding may help to reduce Wnt and β-catenin protein degradation.

The results indicated that GAS stimulated the Wnt/β-catenin signaling pathway within the hippocampal NSPCs of Stress-exposed mice, promoting neurogenesis and stress resilience.

**Fig. 7:**
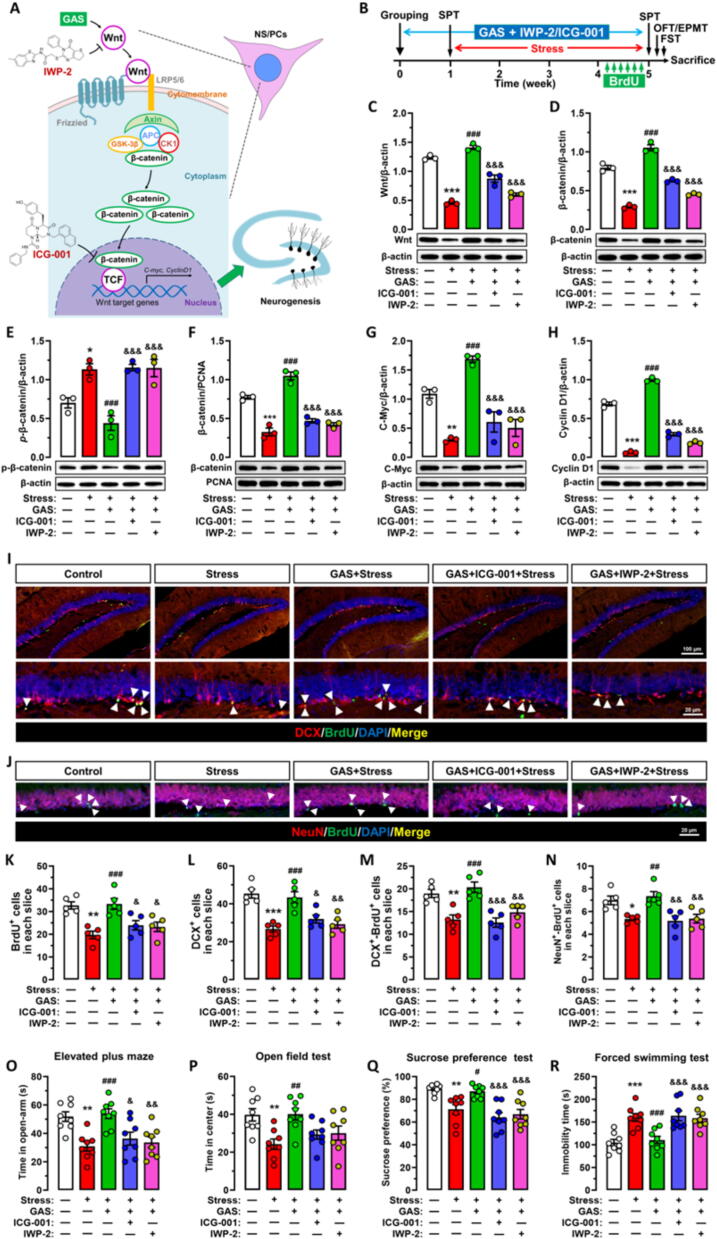
**Blockade of Wnt/β-catenin signaling partially abolished the improvement effects of gastrodin on neurogenesis and behaviors in stress-exposed mice (A-B)** Scheme for blocking the Wnt/β-catenin signaling with IWP-2 or ICG-001 in stress-exposed mice in present of Gastrodin (GAS) treatment. SPT, sucrose preference test; OFT, open field test; EPMT, elevated plus maze test; FST, forced swimming test. **(C**–**H)** Effects of Wnt/β-catenin signaling blockade by IWP-2 or ICG-001 by IWP-2 or ICG-001 on levels of Wnt, β-catenin, *p*-β-catenin, C-Myc and Cyclin D1 in hippocampus of GAS + Stress mice. **(I-N)** Effects of Wnt/β-catenin signaling blockade by IWP-2 or ICG-001 on the hippocampal neurogenesis of stress-exposed mice treated with GAS. Proliferating NSPCs were labeled with BrdU (green), and immature neurons were labeled with Doublecortin (DCX), mature neurons were labeled with NeuN, the newborn immature neurons differentiated from NSPCs were labeled both with DCX and BrdU, the newborn mature neurons were labeled with both BrdU and NeuN, and their number was quantified. **(O-R)** Effects of Wnt/β-catenin signaling blockade by IWP-2 or ICG-001 on the enhanced stress resilience effects of GAS (n = 8). Data are mean ± standard error of the mean (SEM). *p < 0.05, ^**^p < 0.01, ^***^p < 0.001 vs Control group, ^#^*p* < 0.05, ^###^*p* < 0.001 vs Stress group, ^&^*p* < 0.05, ^&&^*p* < 0.01, ^&&^*p* < 0.01 vs GAS group (one-way ANOVA with Tukey's multiple-comparisons test). Details of the statistical analyses are provided in Supplementary Table 7. (For interpretation of the references to colour in this figure legend, the reader is referred to the web version of this article.)

To verify the involvement of Wnt/β-catenin signaling in the GAS-mediated promotion of neurogenesis and enhancement of stress resilience, we propose two strategies to block the Wnt/β-catenin signaling pathway: one involves inhibiting Wnt expression with IWP-2, and inhibition of the nuclear translocation of β-catenin with ICG-001 **(**Fig. 7**A).** Subsequently, we conducted the aforementioned experiments with the addition of the Wnt inhibitor IWP-2 or the β-catenin inhibitor ICG-001 **(**Fig. 7 **B)**, both of which effectively suppressed the Wnt/β-catenin signaling pathway **(**Fig. 7**C–F)**. Furthermore, we observed inhibition of downstream target genes C-Myc and Cyclin D1 expression **(**Fig. 7**G–H)** in hippocampus of GAS-treated mice. Interestingly, we observed that ICG-001 treatment resulted in an upregulation of p-β-catenin levels, which was attributed to its ability to suppress Wnt/β-catenin signaling by impeding the interaction between β-catenin and CBP (CREB-binding protein). This inhibitory effect may consequently modulate the phosphorylation status of β-catenin, thereby influencing p-β-catenin abundance **(**Fig. 7 **E)**.

Significantly, the inhibition of Wnt/β-catenin signaling resulted in the abolishment of the promoting effects of GAS on adult hippocampal neurogenesis in stress-exposed mice. This was evidenced by a reduction in the counts of BrdU^+^ cells, DCX^+^ cells, BrdU^+^-DCX^+^ cells and BrdU^+^-NeuN^+^ cells within hippocampal SGZ of GAS-treated mice when exposed to IWP-2 or ICG-001 **(**Fig. 7 **I–N)**. The findings indicated that the effects of GAS on promoting neurogenesis are partially reliant on the Wnt/β-catenin signaling pathway.

Moreover, our research showed that the stress-resilience enhancement effects of GAS were abolished by inhibiting Wnt/β-catenin signaling. This is demonstrated by a reduction in the time spent in open-arms during the elevated plus maze test, reduced time in the center area during the open field test, lower sucrose preference, and prolonged periods of immobility during the forced swimming test in GAS-treated mice when present of either IWP-2 or ICG-001 **(**Fig. 7 **O–R)**.

These results suggest that GAS elevates stress resilience through promoting Wnt/β-catenin-dependent neurogenesis.

## Discussion

The disruption of neurogenesis in the hippocampus plays a crucial role in the development of depression [49]. The clinical autopsy findings revealed a decrease in cell proliferation within the DG of the hippocampus among depressed individuals, indicating that hippocampal atrophy may be attributed to diminished neurogenesis and loss of mature neurons [50]. Recent research has demonstrated that selective serotonin reuptake inhibitors (SSRIs), have the potential to counteract this pattern, emphasizing a two-way regulatory interaction between neuronal regeneration and depression [51]. Hippocampal neurogenesis is significantly reduced in several depression model mice, such as CMS, CSDS, and CRS [[52], [53], [54]]. Chronic stress-induced depression, as modeled in mice, also impairs NSPCs proliferation and differentiation into and demonstrates a significant decrease in the quantity of hippocampal BrdU^+^-SOX^+^ and BrdU^+^-DCX^+^ cells. Enhancing hippocampal neurogenesis represents an efficacious approach to enhancing stress resilience. Peripheral administration of brain-derived neurotrophic factor (BDNF) via peripheral routes has shown to stimulate neurogenesis, thereby ameliorating depression-like symptoms in rodent models [55]. It has been observed that the administration of interleukin-4 during periods of stress exerts inhibitory effects on hippocampal neurogenesis while concurrently augmenting stress resilience [56]. A recent study has revealed that targeted gene editing enhances neural regeneration, particularly within the hippocampus of mature rats, subsequently resulting in heightened emotional stability and improved quality of life under chronic stress conditions [57]. In our study, we observed that chronic stress did not exert a significant impact on the quantities of BrdU^+^ and DCX^+^-BrdU^+^ cells in present of GAS treatment, suggesting that GAS effectively counteracts the inhibitory effects of chronic stress on the proliferation and differentiation processes of NSPCs.

Adult hippocampal neurogenesis plays a pivotal role in maintaining stress resilience [58]. Blocking adult hippocampal neurogenesis via abrogation methods such as TMZ treatment or irradiation can impair the stress resilience of mice, making them more susceptible to developing depression-like behaviors when exposed to chronic stress [52,59]. Our data demonstrated that TMZ treatment notably diminished the quantity of BrdU^+^, DCX^+^, and DCX^+^-BrdU^+^ cells, suggesting that TMZ blocked the hippocampal neurogenesis. Surprisingly, following TMZ-induced ablation of neurogenesis, stress-exposed mice treated with GAS displayed various negative behavioral features, such as anxiety, anhedonia, and behavioral despair. The findings indicate that the stress-resilience enhancing effects of GAS are partly reliant on the promotion of hippocampal neurogenesis. Future studies can focus on uncovering the specific role of GAS in regulating apoptotic signaling pathways to more fully understand its neuroprotective effects.

The Wnt signaling pathway is crucial for the growth and differentiation of neural progenitor cells in the hippocampus. β-catenin, a key effector, interacts with TCF/LEF motifs in the CyclinD1 promoter to promote cell proliferation and differentiation. Upregulation of β-catenin and CyclinD1 activates the Wnt/β-catenin pathway, significantly influencing hippocampal neurogenesis [60,61]. Inhibition of this pathway can block the neurogenic effects [62]. Additionally, activation of the Wnt/β-catenin pathway can prevent chronic stress-induced decrease in hippocampal neurogenesis [63]. Our data showed that Wnt/β-catenin pathway may serve as a potential mechanism through which GAS enhances neurogenesis in the adult hippocampus. Blockade of Wnt/β-catenin signaling partially abolished the improvement effects of GAS on neurogenesis and behaviors in stress-exposed mice. These results suggest that Wnt/β-catenin signaling pathway plays a crucial role in GAS's promotion of neurogenesis and enhancement of stress resilience. Notably, the efficacy of GAS in the treatment of a variety of neurological diseases may be related to its targeting of multiple pathways. Among these, the Wnt/β-catenin signaling pathway is one of the key mechanisms. Previous studies have shown that GAS can improve cognitive dysfunction induced by sleep deprivation in rats by mediating the activation of the Wnt/β-catenin signaling pathway [64]. In addition, GAS could protect mice against lead-induced nerve injury by activating this signaling pathway [65]. However, it is important to note that GAS also acts on other targets and pathways, which collectively contribute to its broad-spectrum neuroprotective effects. Therefore, future studies could further explore the mechanism of GAS action on different neural targets in order to more fully understand its neuroprotective effects and therapeutic potential.

Particularly, in this study, we identified for the first time that GAS promotes the nuclear translocation of Wnt signaling protein β-catenin in NSPCs located in the SGZ of the hippocampus. This finding indicates that GAS may enhance neurogenesis and stress resilience by specifically targeting the Wnt/β-catenin signaling pathway in hippocampal NSPCs. Future studies can further investigate whether GAS reactivates quiescent neural stem cells in the hippocampus via Wnt/β-catenin signaling pathway, potentially offering therapeutic strategies to improve a broader range of mental disorders and neurodegenerative diseases associated with neurogenesis impairments.

## Conclusion

This study shows that GAS enhances stress resilience by promoting Wnt/β-catenin-dependent neurogenesis. GAS offers potential advantages for treating major depressive disorder, including rapid action, good safety, and multi-target mechanisms, making it a promising candidate, especially for resistant depression. However, due to differences in human and mouse neurogenesis, clinical application may vary. Since its active ingredient penetrates the blood–brain barrier, GAS could still be effective in humans. Future clinical trials are needed to confirm its efficacy and safety. Additionally, systemic administration may cause off-target effects, so further experiments will evaluate GAS's targeting specificity and related signaling pathway inhibitors.

## Author Contributions

H.Z., Z.Z., and P.L. contributed equally to this work. H.Z. and Z.Z. wrote the original manuscript. H.Z., M.W., and J.Z. reviewed and polished the manuscript. J.Z. and Z.Z. designed this study. T.Z., C.X., Z.Y., and J.Z. supervised this study. Z.Z., H.Z., P.L., Y.L., H.H., and Y.G. performed all experiments. P.L. performed statistical analyses of the data. The published version of the manuscript has been read and approved by all authors.

## Data Availability Statement

The complete set of raw read data produced in this research has been submitted to the National Center for Biotechnology Information (NCBI) database, associated with the BioProject ID: PRJNA1174085.

Version date:10/12/2024.

## Compliance with Ethics Requirements

This study and included experimental procedures were approved by the Institutional Animal Care and Use Committee of Guizhou University of Traditional Chinese Medicine (20240220001). All animal housing and experiments were conducted in strict accordance with the laboratory animal care and use regime.

## Declaration of competing interest

The authors declare that they have no known competing financial interests or personal relationships that could have appeared to influence the work reported in this paper.
