# Supplementary material for: Gastrodin enhances stress resilience through promoting Wnt/β-Catenin-dependent neurogenesis
Source: J Adv Res. 2025 Apr 13;80:991–1007. doi: 10.1016/j.jare.2025.04.017 (PMC12869260; doi:10.1016/j.jare.2025.04.017)
Supplement: Supplementary Data 1 [file mmc1.docx]

**Supplementary Materials**

| 1 | Supplementary Figure 1 | Effect of GAS on the proportion of Stress-sensitive and Stress-resistant subgroups in mice |
| --- | --- | --- |
| 2 | Supplementary Table 1 | The F value and P value in multiple comparisons of figure 1 |
| 3 | Supplementary Table 2 | The F value and P value in multiple comparisons of figure 2 |
| 4 | Supplementary Table 3 | The F value and P value in multiple comparisons of figure 3 |
| 5 | Supplementary Table 4 | The F value and P value in multiple comparisons of figure 4 |
| 6 | Supplementary Table 5 | The F value and P value in multiple comparisons of figure 5 |
| 7 | Supplementary Table 6 | The F value and P value in multiple comparisons of figure 6 |
| 8 | Supplementary Table 7 | The F value and P value in multiple comparisons of figure 7 |

**
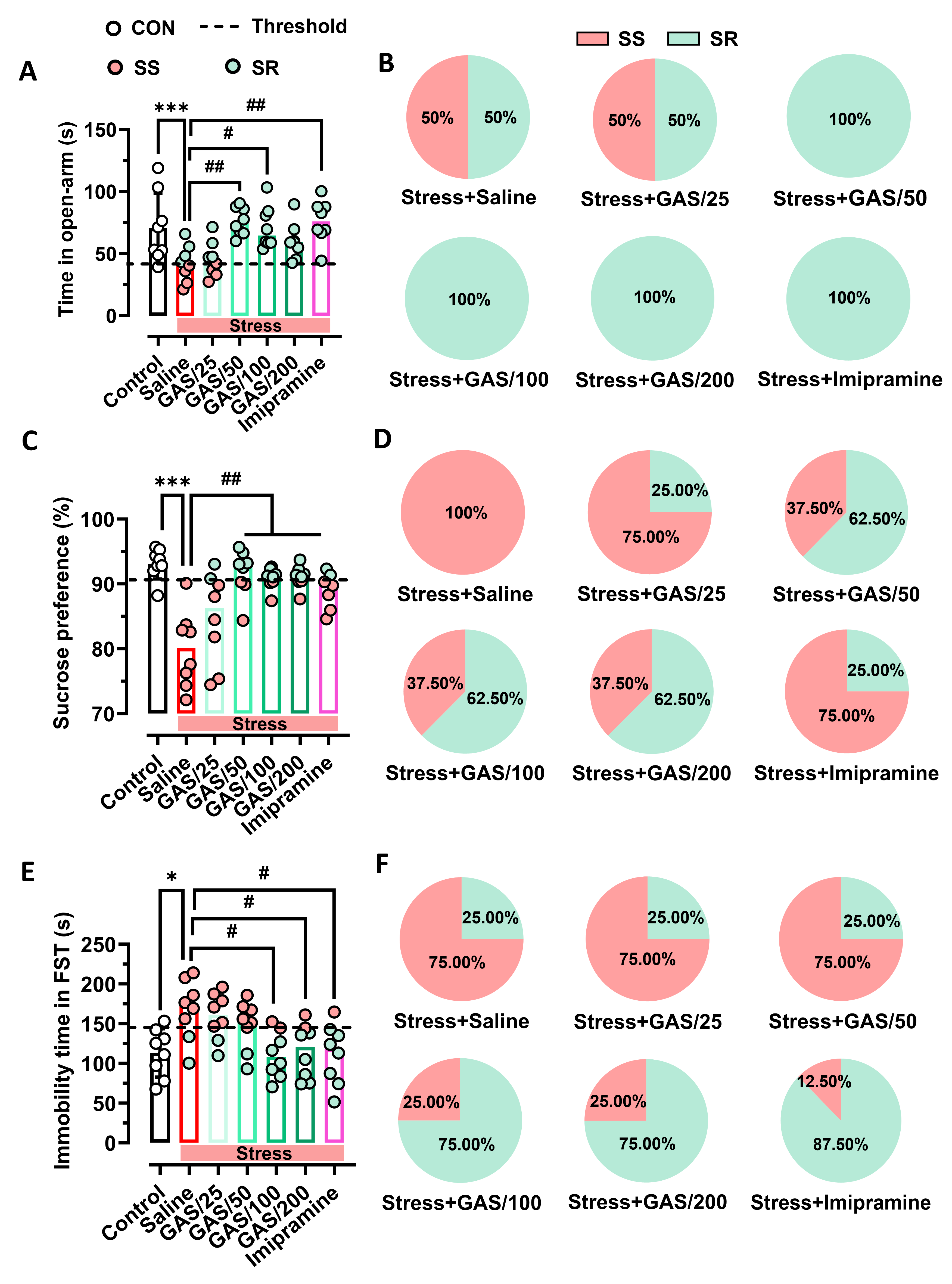
**

**Supplementary Figure S1. Effect of GAS on the proportion of Stress-sensitive and Stress-resistant subgroups in mice**

**(A, C, and E)** The stress-sensitive (SS) subgroup and the stress-resistant (SR) subgroup were identified based on behavioral tests. FST, forced swimming test.

**(B, D, and F)** The proportion of Stress-sensitive and Stress-resistant subgroups was quantified based on behavioral tests.

Data are mean ± standard error of the mean (SEM) (n = 8 mice for each group). ns, not significant, ^*^*p* < 0.05, ^***^*p* < 0.001 vs Control group, ^#^*p* < 0.05, ^##^*p* < 0.01 vs Stress group (one-way ANOVA with Tukey's multiple-comparisons test).

**Supplementary Table 1. The F value and P value in multiple comparisons of figure 1**

| **Marker** | **Groups** | **Mean Diff.** | **Type of ANOVA** | **95.00% CI of diff.** | **F, DFn, Dfd** | **P value** |
| --- | --- | --- | --- | --- | --- | --- |
| *Figure 1E* | GAS/100vs.GAS/50 | 5.800 | One-Way ANOVA  Tukey’s post hoc tests | -1.782to13.38 | F(3,16)=0.4382 | 0.1686 |
|  | GAS/100vs.GAS/10 | 13.00 |  | 5.418to20.58 |  | 0.0008 |
|  | GAS/100vs.GAS/Control | 22.12 |  | 14.54to29.70 |  | <0.0001 |
|  | GAS/50vs.GAS/10 | 7.200 |  | -0.3815to14.78 |  | 0.0657 |
|  | GAS/50vs.GAS/Control | 16.32 |  | 8.738to23.90 |  | <0.0001 |
|  | GAS/10vs.GAS/Control | 9.120 |  | 1.538to16.70 |  | 0.0159 |
| *Figure 1F* | GAS/100vs.GAS/50 | 11.59 | One-Way ANOVA  Tukey’s post hoc tests | -22.97to46.15 | F(3,16)=0.3458 | 0.7739 |
|  | GAS/100vs.GAS/10 | 26.90 |  | -7.665to61.46 |  | 0.1582 |
|  | GAS/100vs.GAS/Control | 49.10 |  | 14.54to83.66 |  | 0.0045 |
|  | GAS/50vs.GAS/10 | 15.31 |  | -19.25to49.87 |  | 0.5954 |
|  | GAS/50vs.GAS/Control | 37.51 |  | 2.947to72.07 |  | 0.0311 |
|  | GAS/10vs.GAS/Control | 22.20 |  | -12.36to56.76 |  | 0.2927 |
| *Figure 1I* | GAS/100vs.GAS/50 | -4.098to9.922 | One-Way ANOVA  Tukey’s post hoc tests | -4.098to9.922 | F(3,16)=0.4221 | 0.6424 |
|  | GAS/100vs.GAS/10 | -0.5435to13.48 |  | -0.5435to13.48 |  | 0.0760 |
|  | GAS/100vs.GAS/Control | 5.112to19.13 |  | 5.112to19.13 |  | 0.0008 |
|  | GAS/50vs.GAS/10 | -3.456to10.56 |  | -3.456to10.56 |  | 0.4881 |
|  | GAS/50vs.GAS/Control | 2.200to16.22 |  | 2.200to16.22 |  | 0.0084 |
|  | GAS/10vs.GAS/Control | -1.354to12.67 |  | -1.354to12.67 |  | 0.1375 |
| *Figure 1K* | GAS/100vs.GAS/50 | 0.5000 | One-Way ANOVA  Tukey’s post hoc tests | -3.772to4.772 | F(3,16)=0.2507 | 0.9866 |
|  | GAS/100vs.GAS/10 | 5.480 |  | 1.208to9.752 |  | 0.0100 |
|  | GAS/100vs.GAS/Control | 7.420 |  | 3.148to11.69 |  | 0.0007 |
|  | GAS/50vs.GAS/10 | 4.980 |  | 0.7084to9.252 |  | 0.0197 |
|  | GAS/50vs.GAS/Control | 6.920 |  | 2.648to11.19 |  | 0.0014 |
|  | GAS/10vs.GAS/Control | 1.940 |  | -2.332to6.212 |  | 0.5764 |
| *Figure 1M* | GAS/100vs.GAS/50 | -5.188 | One-Way ANOVA  Tukey’s post hoc tests | -18.68to8.303 | F(3,16)=0.07547 | 0.6945 |
|  | GAS/100vs.GAS/10 | -10.44 |  | -23.93to3.055 |  | 0.1618 |
|  | GAS/100vs.GAS/Control | -13.23 |  | -26.72to0.2587 |  | 0.0555 |
|  | GAS/50vs.GAS/10 | -5.248 |  | -18.74to8.243 |  | 0.6870 |
|  | GAS/50vs.GAS/Control | -8.044 |  | -21.53to5.447 |  | 0.3528 |
|  | GAS/10vs.GAS/Control | -2.796 |  | -16.29to10.69 |  | 0.9327 |

**Supplementary Table 2. The F value and P value in multiple comparisons of figure 2**

| **Marker** | **Groups** | **Mean Diff.** | **Type of ANOVA** | **95.00% CI of diff.** | **F, DFn, Dfd** | **P value** |
| --- | --- | --- | --- | --- | --- | --- |
| *Figure 2D* | GAS/100vs.GAS/50 | 0.5610 | One-Way ANOVA  Tukey’s post hoc tests | -3.446to4.568 | F(4,20)=0.6376 | 0.9930 |
|  | GAS/100vs.GAS/25 | 2.179 |  | -1.828to6.186 |  | 0.4981 |
|  | GAS/100vs.GAS/Control | 6.799 |  | 2.792to10.81 |  | 0.0005 |
|  | GAS/100vs.GAS/200 | -0.4090 |  | -4.416to3.598 |  | 0.9979 |
|  | GAS/50vs.GAS/25 | 1.618 |  | -2.389to5.625 |  | 0.7467 |
|  | GAS/50vs.GAS/Control | 6.238 |  | 2.231to10.24 |  | 0.0013 |
|  | GAS/50vs.GAS/200 | -0.9700 |  | -4.977to3.037 |  | 0.9483 |
|  | GAS/25vs.GAS/Control | 4.620 |  | 0.6134to8.627 |  | 0.0190 |
|  | GAS/25vs.GAS/200 | -2.588 |  | -6.595to1.419 |  | 0.3330 |
|  | GAS/Controlvs.GAS/200 | -7.208 |  | -11.21to-3.201 |  | 0.0002 |
| *Figure 2F* | GAS/100vs.GAS/50 | 2.100 | One-Way ANOVA  Tukey’s post hoc tests | -1.303to5.503 | F(4,20)=0.7197 | 0.3764 |
|  | GAS/100vs.GAS/25 | 2.778 |  | -0.6255to6.181 |  | 0.1447 |
|  | GAS/100vs.GAS/Control | 3.298 |  | -0.1055to6.701 |  | 0.0604 |
|  | GAS/100vs.GAS/200 | -0.3120 |  | -3.715to3.091 |  | 0.9986 |
|  | GAS/50vs.GAS/25 | 0.6780 |  | -2.725to4.081 |  | 0.9741 |
|  | GAS/50vs.GAS/Control | 1.198 |  | -2.205to4.601 |  | 0.8275 |
|  | GAS/50vs.GAS/200 | -2.412 |  | -5.815to0.9915 |  | 0.2502 |
|  | GAS/25vs.GAS/Control | 0.5200 |  | -2.883to3.923 |  | 0.9903 |
|  | GAS/25vs.GAS/200 | -3.090 |  | -6.493to0.3135 |  | 0.0866 |
|  | GAS/Controlvs.GAS/200 | -3.610 |  | -7.013to-0.2065 |  | 0.0343 |
| *Figure 2H* | GAS/100vs.GAS/50 | 2.454 | One-Way ANOVA  Tukey’s post hoc tests | -3.082to7.990 | F(4,20)=0.4177 | 0.6787 |
|  | GAS/100vs.GAS/25 | 3.586 |  | -1.950to9.122 |  | 0.3304 |
|  | GAS/100vs.GAS/Control | 8.672 |  | 3.136to14.21 |  | 0.0012 |
|  | GAS/100vs.GAS/200 | -0.6900 |  | -6.226to4.846 |  | 0.9955 |
|  | GAS/50vs.GAS/25 | 1.132 |  | -4.404to6.668 |  | 0.9715 |
|  | GAS/50vs.GAS/Control | 6.218 |  | 0.6819to11.75 |  | 0.0230 |
|  | GAS/50vs.GAS/200 | -3.144 |  | -8.680to2.392 |  | 0.4565 |
|  | GAS/25vs.GAS/Control | 5.086 |  | -0.4501to10.62 |  | 0.0814 |
|  | GAS/25vs.GAS/200 | -4.276 |  | -9.812to1.260 |  | 0.1824 |
|  | GAS/Controlvs.GAS/200 | -9.362 |  | -14.90to-3.826 |  | 0.0005 |
| *Figure 2J* | GAS/100vs.GAS/50 | 1.452 | One-Way ANOVA  Tukey’s post hoc tests | -1.394to4.298 | F(4,20)=0.4128 | 0.5581 |
|  | GAS/100vs.GAS/25 | 1.602 |  | -1.244to4.448 |  | 0.4651 |
|  | GAS/100vs.GAS/Control | 1.976 |  | -0.8706to4.822 |  | 0.2680 |
|  | GAS/100vs.GAS/200 | -0.6880 |  | -3.534to2.158 |  | 0.9486 |
|  | GAS/50vs.GAS/25 | 0.1500 |  | -2.696to2.996 |  | 0.9998 |
|  | GAS/50vs.GAS/Control | 0.5236 |  | -2.323to3.370 |  | 0.9806 |
|  | GAS/50vs.GAS/200 | -2.140 |  | -4.986to0.7062 |  | 0.2024 |
|  | GAS/25vs.GAS/Control | 0.3736 |  | -2.473to3.220 |  | 0.9946 |
|  | GAS/25vs.GAS/200 | -2.290 |  | -5.136to0.5562 |  | 0.1541 |
|  | GAS/Controlvs.GAS/200 | -2.664 |  | -5.510to0.1826 |  | 0.0736 |
| *Figure 2L* | GAS/100vs.GAS/25 | 0.4949 | One-Way ANOVA  Tukey’s post hoc tests | -1.653to2.643 | F(4,20)=0.6933 | 0.9565 |
|  | GAS/100vs.GAS/Control | 0.9770 |  | -1.171to3.125 |  | 0.6582 |
|  | GAS/100vs.GAS/200 | 0.08756 |  | -2.061to2.236 |  | >0.9999 |
|  | GAS/50vs.GAS/25 | -0.3618 |  | -2.510to1.786 |  | 0.9860 |
|  | GAS/50vs.GAS/Control | 0.1202 |  | -2.028to2.269 |  | 0.9998 |
|  | GAS/50vs.GAS/200 | -0.7692 |  | -2.917to1.379 |  | 0.8188 |
|  | GAS/25vs.GAS/Control | 0.4821 |  | -1.666to2.630 |  | 0.9603 |
|  | GAS/25vs.GAS/200 | -0.4073 |  | -2.556to1.741 |  | 0.9783 |
|  | GAS/Controlvs.GAS/200 | -0.8894 |  | -3.038to1.259 |  | 0.7295 |
|  | GAS/100vs.GAS/25 | 0.4949 |  | -1.653to2.643 |  | 0.9565 |

**Supplementary Table 3. The F value and P value in multiple comparisons of figure 3**

| **Marker** | | **Groups** | **Mean Diff.** | **Type of ANOVA** | **95.00% CI of diff.** | **F, DFn, Dfd** | **P value** |
| --- | --- | --- | --- | --- | --- | --- | --- |
| *Figure 3B* | Week 0 | Control vs Stress | -0.1875 | Two-Way ANOVA  Tukey’s post hoc tests | -1.629 to 1.254 | F (12, 147)  = 3.559 | 0.9997 |
|  |  | Control vs Stress + GAS/25 | -0.2862 |  | -1.728 to 1.156 |  | 0.9969 |
|  |  | Control vs Stress + GAS/50 | 0.3025 |  | -1.139 to 1.744 |  | 0.9958 |
|  |  | Control vs Stress + GAS/100 | 0.3075 |  | -1.134 to 1.749 |  | 0.9954 |
|  |  | Control vs Stress + GAS/200 | -0.1013 |  | -1.543 to 1.341 |  | >0.9999 |
|  |  | Control vs Stress + IMI | 0.4475 |  | -0.9944 to 1.889 |  | 0.9675 |
|  |  | Stress vs Stress + GAS/25 | -0.09875 |  | -1.541 to 1.343 |  | >0.9999 |
|  |  | Stress vs Stress + GAS/50 | 0.4900 |  | -0.9519 to 1.932 |  | 0.9497 |
|  |  | Stress vs Stress + GAS/100 | 0.4950 |  | -0.9469 to 1.937 |  | 0.9472 |
|  |  | Stress vs Stress + GAS/200 | 0.08625 |  | -1.356 to 1.528 |  | >0.9999 |
|  |  | Stress vs Stress + IMI | 0.6350 |  | -0.8069 to 2.077 |  | 0.8432 |
|  |  | Stress + GAS/25 vs Stress + GAS/50 | 0.5888 |  | -0.8532 to 2.031 |  | 0.8851 |
|  |  | Stress + GAS/25 vs Stress + GAS/100 | 0.5938 |  | -0.8482 to 2.036 |  | 0.8810 |
|  |  | Stress + GAS/25 vs Stress + GAS/200 | 0.1850 |  | -1.257 to 1.627 |  | 0.9997 |
|  |  | Stress + GAS/25 vs Stress + IMI | 0.7337 |  | -0.7082 to 2.176 |  | 0.7316 |
|  |  | Stress + GAS/50 vs Stress + GAS/100 | 0.005000 |  | -1.437 to 1.447 |  | >0.9999 |
|  |  | Stress + GAS/50 vs Stress + GAS/200 | -0.4038 |  | -1.846 to 1.038 |  | 0.9806 |
|  |  | Stress + GAS/50 vs Stress + IMI | 0.1450 |  | -1.297 to 1.587 |  | >0.9999 |
|  |  | Stress + GAS/100 vs Stress + GAS/200 | -0.4088 |  | -1.851 to 1.033 |  | 0.9794 |
|  |  | Stress + GAS/100 vs Stress + IMI | 0.1400 |  | -1.302 to 1.582 |  | >0.9999 |
|  |  | Stress + GAS/200 vs Stress CIMI | 0.5487 |  | -0.8932 to 1.991 |  | 0.9153 |
|  | Week 1 | Control vs Stress | 0.08750 | Two-Way ANOVA  Tukey’s post hoc tests | -1.354 to 1.529 | F (2, 147)  = 5.489 | >0.9999 |
|  |  | Control vs Stress + GAS/25 | 0.1700 |  | -1.272 to 1.612 |  | 0.9998 |
|  |  | Control vs Stress + GAS/50 | 0.8812 |  | -0.5607 to 2.323 |  | 0.5321 |
|  |  | Control vs Stress + GAS/100 | 0.4462 |  | -0.9957 to 1.888 |  | 0.9680 |
|  |  | Control vs Stress + GAS/200 | 0.3812 |  | -1.061 to 1.823 |  | 0.9856 |
|  |  | Control vs Stress + IMI | 0.7475 |  | -0.6944 to 2.189 |  | 0.7142 |
|  |  | Stress vs Stress + GAS/25 | 0.08250 |  | -1.359 to 1.524 |  | >0.9999 |
|  |  | Stress vs Stress + GAS/50 | 0.7937 |  | -0.6482 to 2.236 |  | 0.6530 |
|  |  | Stress vs Stress + GAS/100 | 0.3588 |  | -1.083 to 1.801 |  | 0.9895 |
|  |  | Stress vs Stress + GAS/200 | 0.2937 |  | -1.148 to 1.736 |  | 0.9964 |
|  |  | Stress vs Stress + IMI | 0.6600 |  | -0.7819 to 2.102 |  | 0.8176 |
|  |  | Stress + GAS/25 vs Stress + GAS/50 | 0.7112 |  | -0.7307 to 2.153 |  | 0.7594 |
|  |  | Stress + GAS/25 vs Stress + GAS/100 | 0.2762 |  | -1.166 to 1.718 |  | 0.9975 |
|  |  | Stress + GAS/25 vs Stress + GAS/200 | 0.2112 |  | -1.231 to 1.653 |  | 0.9994 |
|  |  | Stress + GAS/25 vs Stress + IMI | 0.5775 |  | -0.8644 to 2.019 |  | 0.8942 |
|  |  | Stress + GAS/50 vs Stress + GAS/100 | -0.4350 |  | -1.877 to 1.007 |  | 0.9718 |
|  |  | Stress + GAS/50 vs Stress + GAS/200 | -0.5000 |  | -1.942 to 0.9419 |  | 0.9446 |
|  |  | Stress + GAS/50 vs Stress + IMI | -0.1337 |  | -1.576 to 1.308 |  | >0.9999 |
|  |  | Stress + GAS/100 vs Stress + GAS/200 | -0.06500 |  | -1.507 to 1.377 |  | >0.9999 |
|  |  | Stress + GAS/100 vs Stress + IMI | 0.3013 |  | -1.141 to 1.743 |  | 0.9959 |
|  |  | Stress + GAS/200 vs Stress + IMI | 0.3663 |  | -1.076 to 1.808 |  | 0.9883 |
|  | Week 5 | Control vs Stress | 2.799 | Two-Way ANOVA  Tukey’s post hoc tests | 1.357 to 4.241 | F (6, 147)  = 3.705 | <0.0001 |
|  |  | Control vs Stress + GAS/25 | 2.953 |  | 1.511 to 4.394 |  | <0.0001 |
|  |  | Control vs Stress + GAS/50 | 2.271 |  | 0.8293 to 3.713 |  | 0.0001 |
|  |  | Control vs Stress + GAS/100 | 1.064 |  | -0.3782 to 2.506 |  | 0.2990 |
|  |  | Control vs Stress + GAS/200 | 1.539 |  | 0.09681 to 2.981 |  | 0.0282 |
|  |  | Control vs Stress + IMI | 1.635 |  | 0.1931 to 3.077 |  | 0.0153 |
|  |  | Stress vs Stress + GAS/25 | 0.1538 |  | -1.288 to 1.596 |  | >0.9999 |
|  |  | Stress vs Stress + GAS/50 | -0.5275 |  | -1.969 to 0.9144 |  | 0.9291 |
|  |  | Stress vs Stress + GAS/100 | -1.735 |  | -3.177 to -0.2931 |  | 0.0078 |
|  |  | Stress vs Stress + GAS/200 | -1.260 |  | -2.702 to 0.1819 |  | 0.1298 |
|  |  | Stress vs Stress + IMI | -1.164 |  | -2.606 to 0.2782 |  | 0.2007 |
|  |  | Stress + GAS/25 vs Stress + GAS/50 | -0.6813 |  | -2.123 to 0.7607 |  | 0.7944 |
|  |  | Stress + GAS/25 vs Stress + GAS/100 | -1.889 |  | -3.331 to -0.4468 |  | 0.0026 |
|  |  | Stress + GAS/25 vs Stress + GAS/200 | -1.414 |  | -2.856 to 0.02819 |  | 0.0586 |
|  |  | Stress + GAS/25 vs Stress + IMI | -1.318 |  | -2.759 to 0.1244 |  | 0.0977 |
|  |  | Stress + GAS/50 vs Stress + GAS/100 | -1.208 |  | -2.649 to 0.2344 |  | 0.1656 |
|  |  | Stress + GAS/50 vs Stress + GAS/200 | -0.7325 |  | -2.174 to 0.7094 |  | 0.7332 |
|  |  | Stress + GAS/50 vs Stress + IMI | -0.6363 |  | -2.078 to 0.8057 |  | 0.8420 |
|  |  | Stress + GAS/100 vs Stress + GAS/200 | 0.4750 |  | -0.9669 to 1.917 |  | 0.9566 |
|  |  | Stress + GAS/100 vs Stress + IMI | 0.5712 |  | -0.8707 to 2.013 |  | 0.8990 |
|  |  | Stress + GAS/200 vs Stress + IMI | 0.09625 |  | 1.346 to 1.538 |  | >0.9999 |
|  | Control | Week 0 vs Week 1 | -0.1300 | Two-Way ANOVA  Tukey’s post hoc tests | -1.272 to 1.012 |  | 0.9608 |
|  |  | Week 0 vs Week 5 | -1.091 |  | -2.233 to 0.05074 |  | 0.0644 |
|  |  | Week 1 vs Week 5 | -0.9613 |  | -2.103 to 0.1807 |  | 0.1175 |
|  | Stress | Week 0 vs Week 1 | 0.1450 | Two-Way ANOVA  Tukey’s post hoc tests | -0.9970 to 1.287 |  | 0.9514 |
|  |  | Week 0 vs Week 5 | 1.895 |  | 0.7530 to 3.037 |  | 0.0004 |
|  |  | Week 1 vs Week 5 | 1.750 |  | 0.6080 to 2.892 |  | 0.0011 |
|  | Stress + GAS/25 | Week 0 vs Week 1 | 0.3263 | Two-Way ANOVA  Tukey’s post hoc tests | -0.8157 to 1.468 |  | 0.7776 |
|  |  | Week 0 vs Week 5 | 2.148 |  | 1.006 to 3.289 |  | <0.0001 |
|  |  | Week 1 vs Week 5 | 1.821 |  | 0.6793 to 2.963 |  | 0.0007 |
|  | Stress + GAS/50 | Week 0 vs Week 1 | 0.4487 | Two-Way ANOVA  Tukey’s post hoc tests | -0.6932 to 1.591 |  | 0.6220 |
|  |  | Week 0 vs Week 5 | 0.8775 |  | -0.2645 to 2.019 |  | 0.1668 |
|  |  | Week 1 vs Week 5 | 0.4288 |  | -0.7132 to 1.571 |  | 0.6481 |
|  | Stress + GAS/100 | Week 0 vs Week 1 | 0.008750 | Two-Way ANOVA  Tukey’s post hoc tests | -1.133 to 1.151 |  | 0.9998 |
|  |  | Week 0 vs Week 5 | -0.3350 |  | -1.477 to 0.8070 |  | 0.7670 |
|  |  | Week 1 vs Week 5 | -0.3438 |  | -1.486 to 0.7982 |  | 0.7564 |
|  | Stress + GAS/200 | Week 0 vs Week 1 | 0.3525 | Two-Way ANOVA  Tukey’s post hoc tests | -0.7895 to 1.494 |  | 0.7456 |
|  |  | Week 0 vs Week 5 | 0.5488 |  | -0.5932 to 1.691 |  | 0.4925 |
|  |  | Week 1 vs Week 5 | 0.1963 |  | -0.9457 to 1.338 |  | 0.9128 |
|  | Stress + IMI | Week 0 vs Week 1 | 0.1700 | Two-Way ANOVA  Tukey’s post hoc tests | -0.9720 to 1.312 |  | 0.9338 |
|  |  | Week 0 vs Week 5 | 0.09625 |  | -1.046 to 1.238 |  | 0.9783 |
|  |  | Week 1 vs Week 5 | -0.07375 |  | -1.216 to 1.068 |  | 0.9872 |
| *Figure 3C* | | Control vs. Saline | 2.125 | One-Way ANOVA  Tukey’s post hoc tests | 0.3718 to 3.878 | F (6, 49) = 1.544 | 0.0085 |
|  |  | Control vs. GAS/25 | 2.000 |  | 0.2468 to 3.753 |  | 0.0159 |
|  |  | Control vs. GAS/50 | 1.250 |  | -0.5032 to 3.003 |  | 0.3186 |
|  |  | Control vs. GAS/100 | 0.2500 |  | -1.503 to 2.003 |  | 0.9994 |
|  |  | Control vs. GAS/200 | 0.3750 |  | -1.378 to 2.128 |  | 0.9943 |
|  |  | Control vs. Imipramine | 1.000 |  | -0.7532 to 2.753 |  | 0.5847 |
|  |  | Saline vs. GAS/25 | -0.1250 |  | -1.878 to 1.628 |  | >0.9999 |
|  |  | Saline vs. GAS/50 | -0.8750 |  | -2.628 to 0.8782 |  | 0.7232 |
|  |  | Saline vs. GAS/100 | -1.875 |  | -3.628 to -0.1218 |  | 0.0289 |
|  |  | Saline vs. GAS/200 | -1.750 |  | -3.503 to 0.003157 |  | 0.0507 |
|  |  | Saline vs. Imipramine | -1.125 |  | -2.878 to 0.6282 |  | 0.4446 |
|  |  | GAS/25 vs. GAS/50 | -0.7500 |  | -2.503 to 1.003 |  | 0.8416 |
|  |  | GAS/25 vs. GAS/100 | -1.750 |  | -3.503 to 0.003157 |  | 0.0507 |
|  |  | GAS/25 vs. GAS/200 | -1.625 |  | -3.378 to 0.1282 |  | 0.0858 |
|  |  | GAS/25 vs. Imipramine | -1.000 |  | -2.753 to 0.7532 |  | 0.5847 |
|  |  | GAS/50 vs. GAS/100 | -1.000 |  | -2.753 to 0.7532 |  | 0.5847 |
|  |  | GAS/50 vs. GAS/200 | -0.8750 |  | -2.628 to 0.8782 |  | 0.7232 |
|  |  | GAS/50 vs. Imipramine | -0.2500 |  | -2.003 to 1.503 |  | 0.9994 |
|  |  | GAS/100 vs. GAS/200 | 0.1250 |  | -1.628 to 1.878 |  | >0.9999 |
|  |  | GAS/100 vs. Imipramine | 0.7500 |  | -1.003 to 2.503 |  | 0.8416 |
|  |  | GAS/200 vs. Imipramine | 0.6250 |  | -1.128 to 2.378 |  | 0.9263 |
| *Figure 3D* | | Control vs. Saline | -76.13 | One-Way ANOVA  Tukey’s post hoc tests | -144.4 to -7.864 | F (6, 49) = 0.8691 | 0.0198 |
|  |  | Control vs. GAS/25 | -55.13 |  | -123.4 to 13.14 |  | 0.1882 |
|  |  | Control vs. GAS/50 | 7.750 |  | -60.51 to 76.01 |  | 0.9998 |
|  |  | Control vs. GAS/100 | -27.50 |  | -95.76 to 40.76 |  | 0.8755 |
|  |  | Control vs. GAS/200 | -13.00 |  | -81.26 to 55.26 |  | 0.9970 |
|  |  | Control vs. Imipramine | 0.1250 |  | -68.14 to 68.39 |  | >0.9999 |
|  |  | Saline vs. GAS/25 | 21.00 |  | -47.26 to 89.26 |  | 0.9629 |
|  |  | Saline vs. GAS/50 | 83.88 |  | 15.61 to 152.1 |  | 0.0073 |
|  |  | Saline vs. GAS/100 | 48.63 |  | -19.64 to 116.9 |  | 0.3197 |
|  |  | Saline vs. GAS/200 | 63.13 |  | -5.136 to 131.4 |  | 0.0871 |
|  |  | Saline vs. Imipramine | 76.25 |  | 7.989 to 144.5 |  | 0.0195 |
|  |  | GAS/25 vs. GAS/50 | 62.88 |  | -5.386 to 131.1 |  | 0.0894 |
|  |  | GAS/25 vs. GAS/100 | 27.63 |  | -40.64 to 95.89 |  | 0.8732 |
|  |  | GAS/25 vs. GAS/200 | 42.13 |  | -26.14 to 110.4 |  | 0.4919 |
|  |  | GAS/25 vs. Imipramine | 55.25 |  | -13.01 to 123.5 |  | 0.1862 |
|  |  | GAS/50 vs. GAS/100 | -35.25 |  | -103.5 to 33.01 |  | 0.6907 |
|  |  | GAS/50 vs. GAS/200 | -20.75 |  | -89.01 to 47.51 |  | 0.9650 |
|  |  | GAS/50 vs. Imipramine | -7.625 |  | -75.89 to 60.64 |  | 0.9999 |
|  |  | GAS/100 vs. GAS/200 | 14.50 |  | -53.76 to 82.76 |  | 0.9945 |
|  |  | GAS/100 vs. Imipramine | 27.63 |  | -40.64 to 95.89 |  | 0.8732 |
|  |  | GAS/200 vs. Imipramine | 13.13 |  | -55.14 to 81.39 |  | 0.9968 |
| *Figure 3F* | | Control vs. Saline | 14.39 | One-Way ANOVA  Tukey’s post hoc tests | 1.018 to 27.77 | F (6, 49) = 0.3063 | 0.0274 |
|  |  | Control vs. GAS/25 | 5.190 |  | -8.185 to 18.57 |  | 0.8935 |
|  |  | Control vs. GAS/50 | 0.8047 |  | -12.57 to 14.18 |  | >0.9999 |
|  |  | Control vs. GAS/100 | -2.022 |  | -15.40 to 11.35 |  | 0.9992 |
|  |  | Control vs. GAS/200 | 8.345 |  | -5.030 to 21.72 |  | 0.4787 |
|  |  | Control vs. Imipramine | 2.658 |  | -10.72 to 16.03 |  | 0.9962 |
|  |  | Saline vs. GAS/25 | -9.203 |  | -22.58 to 4.173 |  | 0.3604 |
|  |  | Saline vs. GAS/50 | -13.59 |  | -26.96 to -0.2128 |  | 0.0442 |
|  |  | Saline vs. GAS/100 | -16.41 |  | -29.79 to -3.039 |  | 0.0074 |
|  |  | Saline vs. GAS/200 | -6.048 |  | -19.42 to 7.328 |  | 0.8045 |
|  |  | Saline vs. Imipramine | -11.73 |  | -25.11 to 1.640 |  | 0.1207 |
|  |  | GAS/25 vs. GAS/50 | -4.386 |  | -17.76 to 8.990 |  | 0.9498 |
|  |  | GAS/25 vs. GAS/100 | -7.212 |  | -20.59 to 6.163 |  | 0.6465 |
|  |  | GAS/25 vs. GAS/200 | 3.155 |  | -10.22 to 16.53 |  | 0.9903 |
|  |  | GAS/25 vs. Imipramine | -2.532 |  | -15.91 to 10.84 |  | 0.9971 |
|  |  | GAS/50 vs. GAS/100 | -2.827 |  | -16.20 to 10.55 |  | 0.9946 |
|  |  | GAS/50 vs. GAS/200 | 7.541 |  | -5.835 to 20.92 |  | 0.5979 |
|  |  | GAS/50 vs. Imipramine | 1.853 |  | -11.52 to 15.23 |  | 0.9995 |
|  |  | GAS/100 vs. GAS/200 | 10.37 |  | -3.008 to 23.74 |  | 0.2279 |
|  |  | GAS/100 vs. Imipramine | 4.680 |  | -8.696 to 18.06 |  | 0.9323 |
|  |  | GAS/200 vs. Imipramine | -5.687 |  | 1.018 to 27.77 |  | 0.8454 |
| *Figure 3G* | | Control vs. Saline | 40.43 | One-Way ANOVA  Tukey’s post hoc tests | "16.08 to 64.79" | F (6, 49) = 1.309 | 0.0001 |
|  |  | Control vs. GAS/25 | 37.06 |  | "12.71 to 61.41" |  | 0.0004 |
|  |  | Control vs. GAS/50 | 6.479 |  | "-17.87 to 30.83" |  | 0.9820 |
|  |  | Control vs. GAS/100 | 11.47 |  | "-12.88 to 35.83" |  | 0.7731 |
|  |  | Control vs. GAS/200 | 22.50 |  | "-1.850 to 46.86" |  | 0.0875 |
|  |  | Control vs. Imipramine | 6.617 |  | "-17.74 to 30.97" |  | 0.9799 |
|  |  | Saline vs. GAS/25 | -3.374 |  | "-27.73 to 20.98" |  | 0.9995 |
|  |  | Saline vs. GAS/50 | -33.95 |  | "-58.31 to -9.601" |  | 0.0016 |
|  |  | Saline vs. GAS/100 | -28.96 |  | "-53.31 to -4.608" |  | 0.0105 |
|  |  | Saline vs. GAS/200 | -17.93 |  | "-42.28 to 6.423" |  | 0.2824 |
|  |  | Saline vs. Imipramine | -33.82 |  | "-58.17 to -9.463" |  | 0.0016 |
|  |  | GAS/25 vs. GAS/50 | -30.58 |  | "-54.93 to -6.227" |  | 0.0057 |
|  |  | GAS/25 vs. GAS/100 | -25.59 |  | "-49.94 to -1.234" |  | 0.0336 |
|  |  | GAS/25 vs. GAS/200 | -14.56 |  | "-38.91 to 9.798" |  | 0.5303 |
|  |  | GAS/25 vs. Imipramine | -30.44 |  | "-54.79 to -6.088" |  | 0.0061 |
|  |  | GAS/50 vs. GAS/100 | 4.993 |  | "-19.36 to 29.35" |  | 0.9955 |
|  |  | GAS/50 vs. GAS/200 | 16.02 |  | "-8.329 to 40.38" |  | 0.4141 |
|  |  | GAS/50 vs. Imipramine | 0.1386 |  | "-24.21 to 24.49" |  | >0.9999 |
|  |  | GAS/100 vs. GAS/200 | 11.03 |  | "-13.32 to 35.38" |  | 0.8031 |
|  |  | GAS/100 vs. Imipramine | -4.854 |  | "-29.21 to 19.50" |  | 0.9961 |
|  |  | GAS/200 vs. Imipramine | -15.89 |  | "-40.24 to 8.467" |  | 0.4246 |
| *Figure 3H* | Week 0 | Ctrl vs STRESS | 0.8125 | Two-Way ANOVA  Tukey’s post hoc tests | -5.733 to 7.358 | F (12, 147)  = 3.523 | 0.9998 |
|  |  | Ctrl vs STRESS + GAS25 | 1.339 |  | -5.207 to 7.884 |  | 0.9964 |
|  |  | Ctrl vs STRESS + GAS50 | 1.927 |  | -4.618 to 8.473 |  | 0.9750 |
|  |  | Ctrl vs STRESS + GAS100 | 1.558 |  | -4.988 to 8.103 |  | 0.9917 |
|  |  | Ctrl vs STRESS + GAS200 | 1.399 |  | -5.147 to 7.944 |  | 0.9954 |
|  |  | Ctrl vs STRESS + IMI | -0.8025 |  | -7.348 to 5.743 |  | 0.9998 |
|  |  | STRESS vs STRESS + GAS25 | 0.5263 |  | -6.019 to 7.072 |  | >0.9999 |
|  |  | STRESS vs STRESS + GAS50 | 1.115 |  | -5.431 to 7.661 |  | 0.9987 |
|  |  | STRESS vs STRESS + GAS100 | 0.7450 |  | -5.801 to 7.291 |  | 0.9999 |
|  |  | STRESS vs STRESS + GAS200 | 0.5863 |  | -5.959 to 7.132 |  | >0.9999 |
|  |  | STRESS vs STRESS + IMI | -1.615 |  | -8.161 to 4.931 |  | 0.9900 |
|  |  | STRESS + GAS25 vs STRESS + GAS50 | 0.5887 |  | -5.957 to 7.134 |  | >0.9999 |
|  |  | STRESS + GAS25 vs STRESS + GAS100 | 0.2188 |  | -6.327 to 6.764 |  | >0.9999 |
|  |  | STRESS + GAS25 vs STRESS + GAS200 | 0.06000 |  | -6.486 to 6.606 |  | >0.9999 |
|  |  | STRESS + GAS25 vs STRESS + IMI | -2.141 |  | -8.687 to 4.404 |  | 0.9580 |
|  |  | STRESS + GAS50 vs STRESS + GAS100 | -0.3700 |  | -6.916 to 6.176 |  | >0.9999 |
|  |  | STRESS + GAS50 vs STRESS + GAS200 | -0.5287 |  | -7.074 to 6.017 |  | >0.9999 |
|  |  | STRESS + GAS50 vs STRESS + IMI | -2.730 |  | -9.276 to 3.816 |  | 0.8744 |
|  |  | STRESS + GAS100 vs STRESS + GAS200 | -0.1587 |  | -6.704 to 6.387 |  | >0.9999 |
|  |  | STRESS + GAS100 vs STRESS + IMI | -2.360 |  | -8.906 to 4.186 |  | 0.9337 |
|  |  | STRESS + GAS200 vs STRESS + IMI | -2.201 |  | -8.747 to 4.344 |  | 0.9521 |
|  | Week 1 | Ctrl vs STRESS | -0.4358 |  | -6.981 to 6.110 | F (2, 147)  = 1.800 | >0.9999 |
|  |  | Ctrl vs STRESS + GAS25 | -2.120 |  | -8.666 to 4.426 |  | 0.9600 |
|  |  | Ctrl vs STRESS + GAS50 | -2.096 |  | -8.641 to 4.450 |  | 0.9622 |
|  |  | Ctrl vs STRESS + GAS100 | 0.1174 |  | -6.428 to 6.663 |  | >0.9999 |
|  |  | Ctrl vs STRESS + GAS200 | 0.001741 |  | -6.544 to 6.547 |  | >0.9999 |
|  |  | Ctrl vs STRESS + IMI | 0.006180 |  | -6.540 to 6.552 |  | >0.9999 |
|  |  | STRESS vs STRESS + GAS25 | -1.684 |  | -8.230 to 4.862 |  | 0.9875 |
|  |  | STRESS vs STRESS + GAS50 | -1.660 |  | -8.206 to 4.886 |  | 0.9884 |
|  |  | STRESS vs STRESS + GAS100 | 0.5531 |  | -5.993 to 7.099 |  | >0.9999 |
|  |  | STRESS vs STRESS + GAS200 | 0.4375 |  | -6.108 to 6.983 |  | >0.9999 |
|  |  | STRESS vs STRESS + IMI | 0.4420 |  | -6.104 to 6.988 |  | >0.9999 |
|  |  | STRESS + GAS25 vs STRESS + GAS50 | 0.02423 |  | -6.521 to 6.570 |  | >0.9999 |
|  |  | STRESS + GAS25 vs STRESS + GAS100 | 2.237 |  | -4.309 to 8.783 |  | 0.9483 |
|  |  | STRESS + GAS25 vs STRESS + GAS200 | 2.122 |  | -4.424 to 8.667 |  | 0.9599 |
|  |  | STRESS + GAS25 vs STRESS + IMI | 2.126 |  | -4.420 to 8.672 |  | 0.9594 |
|  |  | STRESS + GAS50 vs STRESS + GAS100 | 2.213 |  | -4.333 to 8.759 |  | 0.9509 |
|  |  | STRESS + GAS50 vs STRESS + GAS200 | 2.097 |  | -4.448 to 8.643 |  | 0.9620 |
|  |  | STRESS + GAS50 vs STRESS + IMI | 2.102 |  | -4.444 to 8.647 |  | 0.9616 |
|  |  | STRESS + GAS100 vs STRESS + GAS200 | -0.1156 |  | -6.661 to 6.430 |  | >0.9999 |
|  |  | STRESS + GAS100 vs STRESS + IMI | -0.1112 |  | -6.657 to 6.435 |  | >0.9999 |
|  |  | STRESS + GAS200 vs STRESS + IMI | 0.004439 |  | -6.541 to 6.550 |  | >0.9999 |
|  | Week 5 | Ctrl vs STRESS | 9.921 |  | "3.418 to 16.42" | F (6, 147)  = 2.902 | 0.0002 |
|  |  | Ctrl vs STRESS + GAS25 | 8.393 |  | "1.890 to 14.90" |  | 0.0032 |
|  |  | Ctrl vs STRESS + GAS50 | 1.402 |  | "-5.101 to 7.905" |  | 0.9951 |
|  |  | Ctrl vs STRESS + GAS100 | 1.913 |  | "-4.590 to 8.416" |  | 0.9751 |
|  |  | Ctrl vs STRESS + GAS200 | 2.066 |  | "-4.437 to 8.569" |  | 0.9635 |
|  |  | Ctrl vs STRESS + IMI | 1.743 |  | "-4.760 to 8.246" |  | 0.9845 |
|  |  | STRESS vs STRESS + GAS25 | -1.528 |  | "-8.031 to 4.975" |  | 0.9923 |
|  |  | STRESS vs STRESS + GAS50 | -8.519 |  | "-15.02 to -2.016" |  | 0.0026 |
|  |  | STRESS vs STRESS + GAS100 | -8.009 |  | "-14.51 to -1.506" |  | 0.0059 |
|  |  | STRESS vs STRESS + GAS200 | -7.855 |  | "-14.36 to -1.352" |  | 0.0075 |
|  |  | STRESS vs STRESS + IMI | -8.178 |  | "-14.68 to -1.675" |  | 0.0045 |
|  |  | STRESS + GAS25 vs STRESS + GAS50 | -6.991 |  | "-13.49 to -0.4877" |  | 0.0263 |
|  |  | STRESS + GAS25 vs STRESS + GAS100 | -6.480 |  | "-12.98 to 0.02253" |  | 0.0514 |
|  |  | STRESS + GAS25 vs STRESS + GAS200 | -6.327 |  | "-12.83 to 0.1761" |  | 0.0622 |
|  |  | STRESS + GAS25 vs STRESS + IMI | -6.650 |  | "-13.15 to -0.1471" |  | 0.0414 |
|  |  | STRESS + GAS50 vs STRESS + GAS100 | 0.5103 |  | "-5.993 to 7.013" |  | >0.9999 |
|  |  | STRESS + GAS50 vs STRESS + GAS200 | 0.6638 |  | "-5.839 to 7.167" |  | >0.9999 |
|  |  | STRESS + GAS50 vs STRESS + IMI | 0.3407 |  | "-6.162 to 6.844" |  | >0.9999 |
|  |  | STRESS + GAS100 vs STRESS + GAS200 | 0.1535 |  | "-6.349 to 6.657" |  | >0.9999 |
|  |  | STRESS + GAS100 vs STRESS + IMI | -0.1696 |  | "-6.673 to 6.333" |  | >0.9999 |
|  |  | STRESS + GAS200 vs STRESS + IMI | -0.3232 |  | "-6.826 to 6.180" |  | >0.9999 |
|  | Ctrl | Week 0 vs Week 1 | -0.007260 |  | "-5.157 to 5.143" |  | >0.9999 |
|  |  | Week 0 vs Week 5 | -4.109 |  | "-9.260 to 1.041" |  | 0.1454 |
|  |  | Week 1 vs Week 5 | -4.102 |  | "-9.252 to 1.048" |  | 0.1464 |
|  | STRESS | Week 0 vs Week 1 | -1.256 |  | "-6.406 to 3.895" |  | 0.8325 |
|  |  | Week 0 vs Week 5 | 5.000 |  | "-0.1506 to 10.15" |  | 0.0591 |
|  |  | Week 1 vs Week 5 | 6.255 |  | "1.105 to 11.41" |  | 0.0128 |
|  | STRESS + GAS25 | Week 0 vs Week 1 | -3.466 |  | "-8.616 to 1.684" |  | 0.2517 |
|  |  | Week 0 vs Week 5 | 2.945 |  | "-2.205 to 8.095" |  | 0.3679 |
|  |  | Week 1 vs Week 5 | 6.411 |  | "1.261 to 11.56" |  | 0.0104 |
|  | STRESS + GAS50 | Week 0 vs Week 1 | -4.030 |  | -9.181 to 1.120 |  | 0.1563 |
|  |  | Week 0 vs Week 5 | -4.634 |  | -9.785 to 0.5159 |  | 0.0872 |
|  |  | Week 1 vs Week 5 | -0.6040 |  | -5.754 to 4.546 |  | 0.9584 |
|  | STRESS + GAS100 | Week 0 vs Week 1 | -1.447 |  | -6.598 to 3.703 |  | 0.7839 |
|  |  | Week 0 vs Week 5 | -3.754 |  | -8.904 to 1.396 |  | 0.1990 |
|  |  | Week 1 vs Week 5 | -2.307 |  | -7.457 to 2.844 |  | 0.5401 |
|  | STRESS + GAS200 | Week 0 vs Week 1 | -1.404 |  | -6.554 to 3.746 |  | 0.7952 |
|  |  | Week 0 vs Week 5 | -3.442 |  | -8.592 to 1.708 |  | 0.2565 |
|  |  | Week 1 vs Week 5 | -2.038 |  | -7.188 to 3.113 |  | 0.9279 |
|  | STRESS + IMI | Week 0 vs Week 1 | 0.8014 |  | -4.349 to 5.952 |  | 0.7527 |
|  |  | Week 0 vs Week 5 | -1.564 |  | -6.714 to 3.587 |  | 0.5234 |
|  |  | Week 1 vs Week 5 | -2.365 |  | -7.515 to 2.785 |  | 0.9985 |
| *Figure 3I* | | Control vs. Saline | 23.19 | One-Way ANOVA  Tukey’s post hoc tests | 1.655 to 44.73 | F (6, 49)  =0.7826 | 0.0272 |
|  |  | Control vs. GAS/25 | 15.01 |  | -6.526 to 36.55 |  | 0.3451 |
|  |  | Control vs. GAS/50 | -2.635 |  | -24.17 to 18.90 |  | 0.9998 |
|  |  | Control vs. GAS/100 | -3.236 |  | -24.77 to 18.30 |  | 0.9992 |
|  |  | Control vs. GAS/200 | 2.193 |  | -19.34 to 23.73 |  | >0.9999 |
|  |  | Control vs. Imipramine | 2.966 |  | -18.57 to 24.50 |  | 0.9995 |
|  |  | Saline vs. GAS/25 | -8.181 |  | -29.72 to 13.36 |  | 0.9027 |
|  |  | Saline vs. GAS/50 | -25.83 |  | -47.37 to -4.290 |  | 0.0096 |
|  |  | Saline vs. GAS/100 | -26.43 |  | -47.97 to -4.891 |  | 0.0075 |
|  |  | Saline vs. GAS/200 | -21.00 |  | -42.54 to 0.5384 |  | 0.0604 |
|  |  | Saline vs. Imipramine | -20.23 |  | -41.76 to 1.311 |  | 0.0786 |
|  |  | GAS/25 vs. GAS/50 | -17.65 |  | -39.18 to 3.891 |  | 0.1752 |
|  |  | GAS/25 vs. GAS/100 | -18.25 |  | -39.78 to 3.290 |  | 0.1470 |
|  |  | GAS/25 vs. GAS/200 | -12.82 |  | -34.36 to 8.720 |  | 0.5353 |
|  |  | GAS/25 vs. Imipramine | -12.04 |  | -33.58 to 9.493 |  | 0.6069 |
|  |  | GAS/50 vs. GAS/100 | -0.6010 |  | -22.14 to 20.94 |  | >0.9999 |
|  |  | GAS/50 vs. GAS/200 | 4.829 |  | -16.71 to 26.37 |  | 0.9926 |
|  |  | GAS/50 vs. Imipramine | 5.601 |  | -15.94 to 27.14 |  | 0.9839 |
|  |  | GAS/100 vs. GAS/200 | 5.430 |  | -16.11 to 26.97 |  | 0.9863 |
|  |  | GAS/100 vs. Imipramine | 6.202 |  | -15.34 to 27.74 |  | 0.9731 |
|  |  | GAS/200 vs. Imipramine | 0.7728 |  | -20.76 to 22.31 |  | >0.9999 |
| *Figure 3J* | | Control vs. Saline | 47.08 | One-Way ANOVA  Tukey’s post hoc tests | -440.3 to 534.5 | F (6, 49)  =0.6860 | >0.9999 |
|  |  | Control vs. GAS/25 | 177.1 |  | -310.2 to 664.5 |  | 0.9197 |
|  |  | Control vs. GAS/50 | 46.01 |  | -441.4 to 533.4 |  | >0.9999 |
|  |  | Control vs. GAS/100 | -6.274 |  | -493.7 to 481.1 |  | >0.9999 |
|  |  | Control vs. GAS/200 | 114.6 |  | -372.8 to 601.9 |  | 0.9905 |
|  |  | Control vs. Imipramine | 120.8 |  | -366.6 to 608.2 |  | 0.9875 |
|  |  | Saline vs. GAS/25 | 130.1 |  | -357.3 to 617.5 |  | 0.9817 |
|  |  | Saline vs. GAS/50 | -1.070 |  | -488.5 to 486.3 |  | >0.9999 |
|  |  | Saline vs. GAS/100 | -53.35 |  | -540.7 to 434.0 |  | 0.9999 |
|  |  | Saline vs. GAS/200 | 67.47 |  | -419.9 to 554.9 |  | 0.9995 |
|  |  | Saline vs. Imipramine | 73.69 |  | -413.7 to 561.1 |  | 0.9992 |
|  |  | GAS/25 vs. GAS/50 | -131.1 |  | -618.5 to 356.2 |  | 0.9809 |
|  |  | GAS/25 vs. GAS/100 | -183.4 |  | -670.8 to 304.0 |  | 0.9066 |
|  |  | GAS/25 vs. GAS/200 | -62.60 |  | -550.0 to 424.8 |  | 0.9997 |
|  |  | GAS/25 vs. Imipramine | -56.38 |  | -543.8 to 431.0 |  | 0.9998 |
|  |  | GAS/50 vs. GAS/100 | -52.28 |  | -539.7 to 435.1 |  | 0.9999 |
|  |  | GAS/50 vs. GAS/200 | 68.54 |  | -418.8 to 555.9 |  | 0.9995 |
|  |  | GAS/50 vs. Imipramine | 74.76 |  | -412.6 to 562.1 |  | 0.9991 |
|  |  | GAS/100 vs. GAS/200 | 120.8 |  | -366.6 to 608.2 |  | 0.9875 |
|  |  | GAS/100 vs. Imipramine | 127.0 |  | -360.3 to 614.4 |  | 0.9838 |
|  |  | GAS/200 vs. Imipramine | 6.222 |  | -481.2 to 493.6 |  | >0.9999 |
| *Figure 3L* | | Control vs. Saline | 36.46 | One-Way ANOVA  Tukey’s post hoc tests | 2.507 to 70.42 | F (6, 49)  =0.5160 | 0.0279 |
|  |  | Control vs. GAS/25 | 20.88 |  | -13.08 to 54.83 |  | 0.4965 |
|  |  | Control vs. GAS/50 | 11.33 |  | -22.63 to 45.28 |  | 0.9456 |
|  |  | Control vs. GAS/100 | 0.1000 |  | -33.86 to 34.06 |  | >0.9999 |
|  |  | Control vs. GAS/200 | 9.787 |  | -24.17 to 43.74 |  | 0.9730 |
|  |  | Control vs. Imipramine | 10.49 |  | -23.47 to 44.44 |  | 0.9622 |
|  |  | Saline vs. GAS/25 | -15.59 |  | -49.54 to 18.37 |  | 0.7932 |
|  |  | Saline vs. GAS/50 | -25.14 |  | -59.09 to 8.818 |  | 0.2763 |
|  |  | Saline vs. GAS/100 | -36.36 |  | -70.32 to -2.407 |  | 0.0286 |
|  |  | Saline vs. GAS/200 | -26.68 |  | -60.63 to 7.280 |  | 0.2144 |
|  |  | Saline vs. Imipramine | -25.98 |  | -59.93 to 7.980 |  | 0.2413 |
|  |  | GAS/25 vs. GAS/50 | -9.550 |  | -43.51 to 24.41 |  | 0.9761 |
|  |  | GAS/25 vs. GAS/100 | -20.78 |  | -54.73 to 13.18 |  | 0.5023 |
|  |  | GAS/25 vs. GAS/200 | -11.09 |  | -45.04 to 22.87 |  | 0.9507 |
|  |  | GAS/25 vs. Imipramine | -10.39 |  | -44.34 to 23.57 |  | 0.9639 |
|  |  | GAS/50 vs. GAS/100 | -11.23 |  | -45.18 to 22.73 |  | 0.9478 |
|  |  | GAS/50 vs. GAS/200 | -1.537 |  | -35.49 to 32.42 |  | >0.9999 |
|  |  | GAS/50 vs. Imipramine | -0.8375 |  | -34.79 to 33.12 |  | >0.9999 |
|  |  | GAS/100 vs. GAS/200 | 9.688 |  | -24.27 to 43.64 |  | 0.9744 |
|  |  | GAS/100 vs. Imipramine | 10.39 |  | -23.57 to 44.34 |  | 0.9639 |
|  |  | GAS/200 vs. Imipramine | 0.7000 |  | -33.26 to 34.66 |  | >0.9999 |
| *Figure 3M* | | Control vs. Saline | -54.75 | One-Way ANOVA  Tukey’s post hoc tests | -105.6 to -3.915 | F (6, 49)  =0.2289 | 0.0272 |
|  |  | Control vs. GAS/25 | -45.56 |  | -96.40 to 5.273 |  | 0.1062 |
|  |  | Control vs. GAS/50 | -34.51 |  | -85.35 to 16.32 |  | 0.3763 |
|  |  | Control vs. GAS/100 | 2.287 |  | -48.55 to 53.12 |  | >0.9999 |
|  |  | Control vs. GAS/200 | -1.838 |  | -52.67 to 49.00 |  | >0.9999 |
|  |  | Control vs. Imipramine | 1.662 |  | -49.17 to 52.50 |  | >0.9999 |
|  |  | Saline vs. GAS/25 | 9.188 |  | -41.65 to 60.02 |  | 0.9977 |
|  |  | Saline vs. GAS/50 | 20.24 |  | -30.60 to 71.07 |  | 0.8815 |
|  |  | Saline vs. GAS/100 | 57.04 |  | 6.202 to 107.9 |  | 0.0187 |
|  |  | Saline vs. GAS/200 | 52.91 |  | 2.077 to 103.7 |  | 0.0364 |
|  |  | Saline vs. Imipramine | 56.41 |  | 5.577 to 107.2 |  | 0.0207 |
|  |  | GAS/25 vs. GAS/50 | 11.05 |  | -39.79 to 61.89 |  | 0.9938 |
|  |  | GAS/25 vs. GAS/100 | 47.85 |  | -2.985 to 98.69 |  | 0.0774 |
|  |  | GAS/25 vs. GAS/200 | 43.73 |  | -7.110 to 94.56 |  | 0.1352 |
|  |  | GAS/25 vs. Imipramine | 47.23 |  | -3.610 to 98.06 |  | 0.0845 |
|  |  | GAS/50 vs. GAS/100 | 36.80 |  | -14.04 to 87.64 |  | 0.3013 |
|  |  | GAS/50 vs. GAS/200 | 32.68 |  | -18.16 to 83.51 |  | 0.4425 |
|  |  | GAS/50 vs. Imipramine | 36.18 |  | -14.66 to 87.01 |  | 0.3208 |
|  |  | GAS/100 vs. GAS/200 | -4.125 |  | -54.96 to 46.71 |  | >0.9999 |
|  |  | GAS/100 vs. Imipramine | -0.6250 |  | -51.46 to 50.21 |  | >0.9999 |
|  |  | GAS/200 vs. Imipramine | 3.500 |  | -47.34 to 54.34 |  | >0.9999 |

**Supplementary Table 4. The F value and P value in multiple comparisons of figure 4**

| **Marker** | **Groups** | **Mean Diff.** | **Type of ANOVA** | **95.00% CI of diff.** | **F, DFn, Dfd** | **P value** |
| --- | --- | --- | --- | --- | --- | --- |
| *Figure 4D* | Control vs. Saline | 11.00 | One-Way ANOVA  Tukey’s post hoc tests | 2.636 to 19.36 | F (6, 21) = 0.2737 | 0.0053 |
|  | Control vs. GAS/25 | 11.75 |  | 3.386 to 20.11 |  | 0.0027 |
|  | Control vs. GAS/50 | 0.5000 |  | -7.864 to 8.864 |  | >0.9999 |
|  | Control vs. GAS/100 | -1.830 |  | -10.19 to 6.534 |  | 0.9904 |
|  | Control vs. GAS/200 | -1.805 |  | -10.17 to 6.559 |  | 0.9910 |
|  | Control vs. Imipramine | -0.3625 |  | -8.727 to 8.002 |  | >0.9999 |
|  | Saline vs. GAS/25 | 0.7500 |  | -7.614 to 9.114 |  | >0.9999 |
|  | Saline vs. GAS/50 | -10.50 |  | -18.86 to -2.136 |  | 0.0082 |
|  | Saline vs. GAS/100 | -12.83 |  | -21.19 to -4.466 |  | 0.0010 |
|  | Saline vs. GAS/200 | -12.81 |  | -21.17 to -4.441 |  | 0.0011 |
|  | Saline vs. Imipramine | -11.36 |  | -19.73 to -2.998 |  | 0.0038 |
|  | GAS/25 vs. GAS/50 | -11.25 |  | -19.61 to -2.886 |  | 0.0042 |
|  | GAS/25 vs. GAS/100 | -13.58 |  | -21.94 to -5.216 |  | 0.0005 |
|  | GAS/25 vs. GAS/200 | -13.56 |  | -21.92 to -5.191 |  | 0.0005 |
|  | GAS/25 vs. Imipramine | -12.11 |  | -20.48 to -3.748 |  | 0.0020 |
|  | GAS/50 vs. GAS/100 | -2.330 |  | -10.69 to 6.034 |  | 0.9676 |
|  | GAS/50 vs. GAS/200 | -2.305 |  | -10.67 to 6.059 |  | 0.9692 |
|  | GAS/50 vs. Imipramine | -0.8625 |  | -9.227 to 7.502 |  | 0.9999 |
|  | GAS/100 vs. GAS/200 | 0.02500 |  | -8.339 to 8.389 |  | >0.9999 |
|  | GAS/100 vs. Imipramine | 1.468 |  | -6.897 to 9.832 |  | 0.9971 |
|  | GAS/200 vs. Imipramine | 1.443 |  | 2.636 to 19.36 |  | 0.9973 |
| *Figure 4E* | Control vs. Saline | 6.500 | One-Way ANOVA  Tukey’s post hoc tests | 1.184 to 11.82 | F (6, 21) = 0.3371 | 0.0104 |
|  | Control vs. GAS/25 | 2.538 |  | -2.779 to 7.854 |  | 0.7123 |
|  | Control vs. GAS/50 | -1.083 |  | -6.400 to 4.233 |  | 0.9934 |
|  | Control vs. GAS/100 | 0.1000 |  | -5.216 to 5.416 |  | >0.9999 |
|  | Control vs. GAS/200 | 0.2675 |  | -5.049 to 5.584 |  | >0.9999 |
|  | Control vs. Imipramine | 1.693 |  | -3.624 to 7.009 |  | 0.9397 |
|  | Saline vs. GAS/25 | -3.963 |  | -9.279 to 1.354 |  | 0.2378 |
|  | Saline vs. GAS/50 | -7.583 |  | -12.90 to -2.267 |  | 0.0023 |
|  | Saline vs. GAS/100 | -6.400 |  | -11.72 to -1.084 |  | 0.0119 |
|  | Saline vs. GAS/200 | -6.233 |  | -11.55 to -0.9161 |  | 0.0150 |
|  | Saline vs. Imipramine | -4.808 |  | -10.12 to 0.5089 |  | 0.0936 |
|  | GAS/25 vs. GAS/50 | -3.621 |  | -8.937 to 1.696 |  | 0.3296 |
|  | GAS/25 vs. GAS/100 | -2.438 |  | -7.754 to 2.879 |  | 0.7472 |
|  | GAS/25 vs. GAS/200 | -2.270 |  | -7.586 to 3.046 |  | 0.8020 |
|  | GAS/25 vs. Imipramine | -0.8450 |  | -6.161 to 4.471 |  | 0.9983 |
|  | GAS/50 vs. GAS/100 | 1.183 |  | -4.133 to 6.500 |  | 0.9895 |
|  | GAS/50 vs. GAS/200 | 1.351 |  | -3.966 to 6.667 |  | 0.9793 |
|  | GAS/50 vs. Imipramine | 2.776 |  | -2.541 to 8.092 |  | 0.6250 |
|  | GAS/100 vs. GAS/200 | 0.1675 |  | -5.149 to 5.484 |  | >0.9999 |
|  | GAS/100 vs. Imipramine | 1.593 |  | -3.724 to 6.909 |  | 0.9543 |
|  | GAS/200 vs. Imipramine | 1.425 |  | -3.891 to 6.741 |  | 0.9731 |
| *Figure 4F* | Control vs. Saline | 7.250 | One-Way ANOVA  Tukey’s post hoc tests | 3.072 to 11.43 | F (6, 21) = 1.012 | 0.0002 |
|  | Control vs. GAS/25 | 6.000 |  | 1.822 to 10.18 |  | 0.0021 |
|  | Control vs. GAS/50 | 1.873 |  | -2.305 to 6.050 |  | 0.7656 |
|  | Control vs. GAS/100 | 1.195 |  | -2.983 to 5.373 |  | 0.9632 |
|  | Control vs. GAS/200 | 0.9200 |  | -3.258 to 5.098 |  | 0.9900 |
|  | Control vs. Imipramine | 2.005 |  | -2.173 to 6.183 |  | 0.7073 |
|  | Saline vs. GAS/25 | -1.250 |  | -5.428 to 2.928 |  | 0.9546 |
|  | Saline vs. GAS/50 | -5.378 |  | -9.555 to -1.200 |  | 0.0065 |
|  | Saline vs. GAS/100 | -6.055 |  | -10.23 to -1.877 |  | 0.0019 |
|  | Saline vs. GAS/200 | -6.330 |  | -10.51 to -2.152 |  | 0.0012 |
|  | Saline vs. Imipramine | -5.245 |  | -9.423 to -1.067 |  | 0.0082 |
|  | GAS/25 vs. GAS/50 | -4.128 |  | -8.305 to 0.05043 |  | 0.0542 |
|  | GAS/25 vs. GAS/100 | -4.805 |  | -8.983 to -0.6271 |  | 0.0176 |
|  | GAS/25 vs. GAS/200 | -5.080 |  | -9.258 to -0.9021 |  | 0.0109 |
|  | GAS/25 vs. Imipramine | -3.995 |  | -8.173 to 0.1829 |  | 0.0669 |
|  | GAS/50 vs. GAS/100 | -0.6775 |  | -4.855 to 3.500 |  | 0.9981 |
|  | GAS/50 vs. GAS/200 | -0.9525 |  | -5.130 to 3.225 |  | 0.9881 |
|  | GAS/50 vs. Imipramine | 0.1325 |  | -4.045 to 4.310 |  | >0.9999 |
|  | GAS/100 vs. GAS/200 | -0.2750 |  | -4.453 to 3.903 |  | >0.9999 |
|  | GAS/100 vs. Imipramine | 0.8100 |  | -3.368 to 4.988 |  | 0.9949 |
|  | GAS/200 vs. Imipramine | 1.085 |  | 3.072 to 11.43 |  | 0.9770 |
| *Figure 4G* | Control vs. Saline | 17.58 | One-Way ANOVA  Tukey’s post hoc tests | 1.742 to 33.42 | F (6, 21) = 0.2710 | 0.0234 |
|  | Control vs. GAS/25 | 13.76 |  | -2.082 to 29.60 |  | 0.1169 |
|  | Control vs. GAS/50 | 5.873 |  | -9.966 to 21.71 |  | 0.8844 |
|  | Control vs. GAS/100 | -4.776 |  | -20.61 to 11.06 |  | 0.9529 |
|  | Control vs. GAS/200 | -0.5247 |  | -16.36 to 15.31 |  | >0.9999 |
|  | Control vs. Imipramine | 6.758 |  | -9.081 to 22.60 |  | 0.8025 |
|  | Saline vs. GAS/25 | -3.824 |  | -19.66 to 12.01 |  | 0.9840 |
|  | Saline vs. GAS/50 | -11.71 |  | -27.55 to 4.130 |  | 0.2456 |
|  | Saline vs. GAS/100 | -22.36 |  | -38.20 to -6.518 |  | 0.0026 |
|  | Saline vs. GAS/200 | -18.11 |  | -33.94 to -2.267 |  | 0.0185 |
|  | Saline vs. Imipramine | -10.82 |  | -26.66 to 5.016 |  | 0.3260 |
|  | GAS/25 vs. GAS/50 | -7.884 |  | -23.72 to 7.954 |  | 0.6729 |
|  | GAS/25 vs. GAS/100 | -18.53 |  | -34.37 to -2.694 |  | 0.0152 |
|  | GAS/25 vs. GAS/200 | -14.28 |  | -30.12 to 1.557 |  | 0.0951 |
|  | GAS/25 vs. Imipramine | -6.999 |  | -22.84 to 8.840 |  | 0.7767 |
|  | GAS/50 vs. GAS/100 | -10.65 |  | -26.49 to 5.190 |  | 0.3437 |
|  | GAS/50 vs. GAS/200 | -6.397 |  | -22.24 to 9.442 |  | 0.8385 |
|  | GAS/50 vs. Imipramine | 0.8852 |  | -14.95 to 16.72 |  | >0.9999 |
|  | GAS/100 vs. GAS/200 | 4.251 |  | -11.59 to 20.09 |  | 0.9729 |
|  | GAS/100 vs. Imipramine | 11.53 |  | -4.305 to 27.37 |  | 0.2603 |
|  | GAS/200 vs. Imipramine | 7.283 |  | -8.556 to 23.12 |  | 0.7448 |
| *Figure 4K* | Control vs. Saline | 4.830 | One-Way ANOVA  Tukey’s post hoc tests | 1.343 to 8.317 | F (6, 21) = 0.7548 | 0.0031 |
|  | Control vs. GAS/25 | 4.465 |  | 0.9777 to 7.952 |  | 0.0068 |
|  | Control vs. GAS/50 | 1.933 |  | -1.555 to 5.420 |  | 0.5613 |
|  | Control vs. GAS/100 | -1.070 |  | -4.557 to 2.417 |  | 0.9490 |
|  | Control vs. GAS/200 | 0.1100 |  | -3.377 to 3.597 |  | >0.9999 |
|  | Control vs. Imipramine | 1.168 |  | -2.320 to 4.655 |  | 0.9247 |
|  | Saline vs. GAS/25 | -0.3650 |  | -3.852 to 3.122 |  | 0.9998 |
|  | Saline vs. GAS/50 | -2.898 |  | -6.385 to 0.5898 |  | 0.1468 |
|  | Saline vs. GAS/100 | -5.900 |  | -9.387 to -2.413 |  | 0.0003 |
|  | Saline vs. GAS/200 | -4.720 |  | -8.207 to -1.233 |  | 0.0040 |
|  | Saline vs. Imipramine | -3.663 |  | -7.150 to -0.1752 |  | 0.0355 |
|  | GAS/25 vs. GAS/50 | -2.533 |  | -6.020 to 0.9548 |  | 0.2630 |
|  | GAS/25 vs. GAS/100 | -5.535 |  | -9.022 to -2.048 |  | 0.0007 |
|  | GAS/25 vs. GAS/200 | -4.355 |  | -7.842 to -0.8677 |  | 0.0086 |
|  | GAS/25 vs. Imipramine | -3.298 |  | -6.785 to 0.1898 |  | 0.0717 |
|  | GAS/50 vs. GAS/100 | -3.003 |  | -6.490 to 0.4848 |  | 0.1225 |
|  | GAS/50 vs. GAS/200 | -1.823 |  | -5.310 to 1.665 |  | 0.6240 |
|  | GAS/50 vs. Imipramine | -0.7650 |  | -4.252 to 2.722 |  | 0.9902 |
|  | GAS/100 vs. GAS/200 | 1.180 |  | -2.307 to 4.667 |  | 0.9211 |
|  | GAS/100 vs. Imipramine | 2.238 |  | -1.250 to 5.725 |  | 0.3956 |
|  | GAS/200 vs. Imipramine | 1.058 |  | -2.430 to 4.545 |  | 0.9517 |
| *Figure 4L* | Control vs. Saline | 2.963 | One-Way ANOVA  Tukey’s post hoc tests | 0.4448 to 5.480 | F (6, 21) = 0.8555 | 0.0145 |
|  | Control vs. GAS/25 | 2.208 |  | -0.3102 to 4.725 |  | 0.1111 |
|  | Control vs. GAS/50 | 0.9975 |  | -1.520 to 3.515 |  | 0.8499 |
|  | Control vs. GAS/100 | -0.3400 |  | -2.858 to 2.178 |  | 0.9993 |
|  | Control vs. GAS/200 | -0.07250 |  | -2.590 to 2.445 |  | >0.9999 |
|  | Control vs. Imipramine | 0.1550 |  | -2.363 to 2.673 |  | >0.9999 |
|  | Saline vs. GAS/25 | -0.7550 |  | -3.273 to 1.763 |  | 0.9541 |
|  | Saline vs. GAS/50 | -1.965 |  | -4.483 to 0.5527 |  | 0.1963 |
|  | Saline vs. GAS/100 | -3.303 |  | -5.820 to -0.7848 |  | 0.0054 |
|  | Saline vs. GAS/200 | -3.035 |  | -5.553 to -0.5173 |  | 0.0118 |
|  | Saline vs. Imipramine | -2.808 |  | -5.325 to -0.2898 |  | 0.0225 |
|  | GAS/25 vs. GAS/50 | -1.210 |  | -3.728 to 1.308 |  | 0.7060 |
|  | GAS/25 vs. GAS/100 | -2.548 |  | -5.065 to -0.02975 |  | 0.0462 |
|  | GAS/25 vs. GAS/200 | -2.280 |  | -4.798 to 0.2377 |  | 0.0928 |
|  | GAS/25 vs. Imipramine | -2.053 |  | -4.570 to 0.4652 |  | 0.1609 |
|  | GAS/50 vs. GAS/100 | -1.338 |  | -3.855 to 1.180 |  | 0.6069 |
|  | GAS/50 vs. GAS/200 | -1.070 |  | -3.588 to 1.448 |  | 0.8053 |
|  | GAS/50 vs. Imipramine | -0.8425 |  | -3.360 to 1.675 |  | 0.9249 |
|  | GAS/100 vs. GAS/200 | 0.2675 |  | -2.250 to 2.785 |  | 0.9998 |
|  | GAS/100 vs. Imipramine | 0.4950 |  | -2.023 to 3.013 |  | 0.9945 |
|  | GAS/200 vs. Imipramine | 0.2275 |  | -2.290 to 2.745 |  | >0.9999 |

**Supplementary Table 5. The F value and P value in multiple comparisons of figure 5**

| **Marker** | **Groups** | **Mean Diff.** | **Type of ANOVA** | **95.00% CI of diff.** | **F, DFn, Dfd** | **P value** |
| --- | --- | --- | --- | --- | --- | --- |
| *Figure 5D* | Ctrl vs. Stress | 9.000 | One-Way ANOVA  Tukey’s post hoc tests | 2.673 to 15.33 | F (3, 16) = 0.3176 | 0.0044 |
|  | Ctrl vs. Stress + GAS | 1.000 |  | -5.327 to 7.327 |  | 0.9682 |
|  | Ctrl vs. TMZ | 12.80 |  | 6.473 to 19.13 |  | 0.0001 |
|  | Stress vs. Stress + GAS | -8.000 |  | -14.33 to -1.673 |  | 0.0111 |
|  | Stress vs. TMZ | 3.800 |  | -2.527 to 10.13 |  | 0.3468 |
|  | Stress + GAS vs. TMZ | 15.60 |  | 5.473 to 18.13 |  | 0.0004 |
| *Figure 5E* | Ctrl vs. STRESS | 14.00 | One-Way ANOVA  Tukey’s post hoc tests | 5.089 to 22.91 | F (3, 16) = 0.1433 | 0.0019 |
|  | Ctrl vs. STRESS + GAS | -1.800 |  | -10.71 to 7.111 |  | 0.9373 |
|  | Ctrl vs. H-89 | 12.60 |  | 3.689 to 21.51 |  | 0.0047 |
|  | STRESS vs. STRESS + GAS | -15.80 |  | -24.71 to -6.889 |  | 0.0006 |
|  | STRESS vs. H-89 | -1.400 |  | -10.31 to 7.511 |  | 0.9688 |
|  | STRESS + GAS vs. H-89 | 14.40 |  | 5.489 to 23.31 |  | 0.0014 |
| *Figure 5F* | Ctrl vs. STRESS | 5.000 | One-Way ANOVA  Tukey’s post hoc tests | 1.999 to 8.001 | F (3, 16) = 0.1222 | 0.0011 |
|  | Ctrl vs. STRESS + GAS | -0.8000 |  | -3.801 to 2.201 |  | 0.8699 |
|  | Ctrl vs. H-89 | 9.400 |  | 6.399 to 12.40 |  | 0.0001 |
|  | STRESS vs. STRESS + GAS | -5.800 |  | -8.801 to -2.799 |  | 0.0002 |
|  | STRESS vs. H-89 | 4.400 |  | 1.399 to 7.401 |  | 0.0034 |
|  | STRESS + GAS vs. H-89 | 10.20 |  | 7.199 to 13.20 |  | 0.0001 |
| *Figure 5G* | Ctrl vs. STRESS | 0.6000 | One-Way ANOVA  Tukey’s post hoc tests | -1.502 to 2.702 | F (3, 16) = 0.4103 | 0.8458 |
|  | Ctrl vs. STRESS + GAS | 1.000 |  | -1.102 to 3.102 |  | 0.5401 |
|  | Ctrl vs. H-89 | 3.400 |  | 1.298 to 5.502 |  | 0.0014 |
|  | STRESS vs. STRESS + GAS | 0.4000 |  | -1.702 to 2.502 |  | 0.9467 |
|  | STRESS vs. H-89 | 2.800 |  | 0.6976 to 4.902 |  | 0.0075 |
|  | STRESS + GAS vs. H-89 | 2.400 |  | 0.2976 to 4.502 |  | 0.0226 |
| *Figure 5I* | Ctrl vs. STRESS | 26.20 | One-Way ANOVA  Tukey’s post hoc tests | 0.7124 to 51.69 | F (3, 35) = 1.491 | 0.0420 |
|  | Ctrl vs. STRESS + GAS | -1.656 |  | -27.15 to 23.83 |  | 0.9981 |
|  | Ctrl vs. H-89 | 28.48 |  | 2.289 to 54.67 |  | 0.0287 |
|  | STRESS vs. STRESS + GAS | -27.86 |  | -53.35 to -2.368 |  | 0.0277 |
|  | STRESS vs. H-89 | 2.275 |  | -23.91 to 28.46 |  | 0.9954 |
|  | STRESS + GAS vs. H-89 | 30.13 |  | 3.945 to 56.32 |  | 0.0189 |
| *Figure 5K* | Ctrl vs. STRESS | 10.40 | One-Way ANOVA  Tukey’s post hoc tests | 1.722 to 19.08 | F (3, 36) = 1.154 | 0.0135 |
|  | Ctrl vs. STRESS + GAS | 0.5454 |  | -8.131 to 9.222 |  | 0.9982 |
|  | Ctrl vs. H-89 | 16.42 |  | 7.745 to 25.10 |  | <0.0001 |
|  | STRESS vs. STRESS + GAS | -9.853 |  | -18.53 to -1.176 |  | 0.0208 |
|  | STRESS vs. H-89 | 6.024 |  | -2.653 to 14.70 |  | 0.2588 |
|  | STRESS + GAS vs. H-89 | 15.88 |  | 7.200 to 24.55 |  | 0.0001 |
| *Figure 5L* | Control vs. STRESS | 10.34 | One-Way ANOVA  Tukey’s post hoc tests | 0.5208 to 20.16 | F (3, 36) = 1.019 | 0.0358 |
|  | Control vs. STRESS + GAS | -4.285 |  | -14.10 to 5.534 |  | 0.6460 |
|  | Control vs. H-89 | 15.53 |  | 5.710 to 25.35 |  | 0.0008 |
|  | STRESS vs. STRESS + GAS | -14.62 |  | -24.44 to -4.805 |  | 0.0016 |
|  | STRESS vs. H-89 | 5.189 |  | -4.629 to 15.01 |  | 0.4934 |
|  | STRESS + GAS vs. H-89 | 19.81 |  | 9.995 to 29.63 |  | 0.0001 |
| *Figure 5N* | Ctrl vs. STRESS | 20.55 | One-Way ANOVA  Tukey’s post hoc tests | 1.720 to 39.38 | F (3, 36) = 0.3662 | 0.0279 |
|  | Ctrl vs. STRESS + GAS | -2.521 |  | -21.35 to 16.31 |  | 0.9837 |
|  | Ctrl vs. H-89 | 26.98 |  | 8.150 to 45.81 |  | 0.0025 |
|  | STRESS vs. STRESS + GAS | -23.07 |  | -41.90 to -4.241 |  | 0.0112 |
|  | STRESS vs. H-89 | 6.430 |  | -12.40 to 25.26 |  | 0.7945 |
|  | STRESS + GAS vs. H-89 | 29.50 |  | 10.67 to 48.33 |  | 0.0009 |
| *Figure 5O* | Ctrl vs. STRESS | -70.15 | One-Way ANOVA  Tukey’s post hoc tests | -110.1 to -30.24 | F (3, 36) = 0.3305 | 0.0002 |
|  | Ctrl vs. STRESS + GAS | -11.02 |  | -50.93 to 28.89 |  | 0.8787 |
|  | Ctrl vs. H-89 | -51.42 |  | -91.33 to -11.51 |  | 0.0072 |
|  | STRESS vs. STRESS + GAS | 59.13 |  | 19.22 to 99.04 |  | 0.0017 |
|  | STRESS vs. H-89 | 18.73 |  | -21.18 to 58.64 |  | 0.5912 |
|  | STRESS + GAS vs. H-89 | -40.40 |  | -80.31 to -0.4896 |  | 0.0463 |

**Supplementary Table 6. The F value and P value in multiple comparisons of figure6**

| **Marker** | **Groups** | **Mean Diff.** | **Type of ANOVA** | **95.00% CI of diff.** | **F, DFn, Dfd** | **P value** |
| --- | --- | --- | --- | --- | --- | --- |
| *Figure 6E* | Control vs. Saline | 0.4800 | One-Way ANOVA  Tukey’s post hoc tests | 0.3657 to 0.5943 | F (6, 14) = 0.2052 | <0.0001 |
|  | Control vs. GAS/25 | 0.2800 |  | 0.1657 to 0.3943 |  | <0.0001 |
|  | Control vs. GAS/50 | 0.003333 |  | -0.1110 to 0.1176 |  | >0.9999 |
|  | Control vs. GAS/100 | -0.3533 |  | -0.4676 to -0.2390 |  | <0.0001 |
|  | Control vs. GAS/200 | -0.3833 |  | -0.4976 to -0.2690 |  | <0.0001 |
|  | Control vs. Imipramine | 0.3400 |  | 0.2257 to 0.4543 |  | <0.0001 |
|  | Saline vs. GAS/25 | -0.2000 |  | -0.3143 to -0.08569 |  | 0.0005 |
|  | Saline vs. GAS/50 | -0.4767 |  | -0.5910 to -0.3624 |  | <0.0001 |
|  | Saline vs. GAS/100 | -0.8333 |  | -0.9476 to -0.7190 |  | <0.0001 |
|  | Saline vs. GAS/200 | -0.8633 |  | -0.9776 to -0.7490 |  | <0.0001 |
|  | Saline vs. Imipramine | -0.1400 |  | -0.2543 to -0.02569 |  | 0.0125 |
|  | GAS/25 vs. GAS/50 | -0.2767 |  | -0.3910 to -0.1624 |  | <0.0001 |
|  | GAS/25 vs. GAS/100 | -0.6333 |  | -0.7476 to -0.5190 |  | <0.0001 |
|  | GAS/25 vs. GAS/200 | -0.6633 |  | -0.7776 to -0.5490 |  | <0.0001 |
|  | GAS/25 vs. Imipramine | 0.06000 |  | -0.05431 to 0.1743 |  | 0.5728 |
|  | GAS/50 vs. GAS/100 | -0.3567 |  | -0.4710 to -0.2424 |  | <0.0001 |
|  | GAS/50 vs. GAS/200 | -0.3867 |  | -0.5010 to -0.2724 |  | <0.0001 |
|  | GAS/50 vs. Imipramine | 0.3367 |  | 0.2224 to 0.4510 |  | <0.0001 |
|  | GAS/100 vs. GAS/200 | -0.03000 |  | -0.1443 to 0.08431 |  | 0.9672 |
|  | GAS/100 vs. Imipramine | 0.6933 |  | 0.5790 to 0.8076 |  | <0.0001 |
|  | GAS/200 vs. Imipramine | 0.7233 |  | 0.6090 to 0.8376 |  | <0.0001 |
| *Figure 6F* | Control vs. Saline | 0.9400 | One-Way ANOVA  Tukey’s post hoc tests | 0.6054 to 1.275 | F (6, 14) = 0.4184 | <0.0001 |
|  | Control vs. GAS/25 | 0.5567 |  | 0.2221 to 0.8913 |  | 0.0009 |
|  | Control vs. GAS/50 | -0.01000 |  | -0.3446 to 0.3246 |  | >0.9999 |
|  | Control vs. GAS/100 | -0.1633 |  | -0.4979 to 0.1713 |  | 0.6461 |
|  | Control vs. GAS/200 | -0.1233 |  | -0.4579 to 0.2113 |  | 0.8593 |
|  | Control vs. Imipramine | 0.5433 |  | 0.2087 to 0.8779 |  | 0.0011 |
|  | Saline vs. GAS/25 | -0.3833 |  | -0.7179 to -0.04872 |  | 0.0204 |
|  | Saline vs. GAS/50 | -0.9500 |  | -1.285 to -0.6154 |  | <0.0001 |
|  | Saline vs. GAS/100 | -1.103 |  | -1.438 to -0.7687 |  | <0.0001 |
|  | Saline vs. GAS/200 | -1.063 |  | -1.398 to -0.7287 |  | <0.0001 |
|  | Saline vs. Imipramine | -0.3967 |  | -0.7313 to -0.06205 |  | 0.0159 |
|  | GAS/25 vs. GAS/50 | -0.5667 |  | -0.9013 to -0.2321 |  | 0.0007 |
|  | GAS/25 vs. GAS/100 | -0.7200 |  | -1.055 to -0.3854 |  | <0.0001 |
|  | GAS/25 vs. GAS/200 | -0.6800 |  | -1.015 to -0.3454 |  | 0.0001 |
|  | GAS/25 vs. Imipramine | -0.01333 |  | -0.3479 to 0.3213 |  | >0.9999 |
|  | GAS/50 vs. GAS/100 | -0.1533 |  | -0.4879 to 0.1813 |  | 0.7048 |
|  | GAS/50 vs. GAS/200 | -0.1133 |  | -0.4479 to 0.2213 |  | 0.8991 |
|  | GAS/50 vs. Imipramine | 0.5533 |  | 0.2187 to 0.8879 |  | 0.0009 |
|  | GAS/100 vs. GAS/200 | 0.04000 |  | -0.2946 to 0.3746 |  | 0.9995 |
|  | GAS/100 vs. Imipramine | 0.7067 |  | 0.3721 to 1.041 |  | <0.0001 |
|  | GAS/200 vs. Imipramine | 0.6667 |  | 0.3321 to 1.001 |  | 0.0001 |
| *Figure 6G* | Control vs. Saline | -0.3767 | One-Way ANOVA  Tukey’s post hoc tests | -0.6081 to -0.1452 | F (6, 14) = 0.8410 | 0.0011 |
|  | Control vs. GAS/25 | -0.1533 |  | -0.3848 to 0.07810 |  | 0.3249 |
|  | Control vs. GAS/50 | 0.1933 |  | -0.03810 to 0.4248 |  | 0.1318 |
|  | Control vs. GAS/100 | 0.4067 |  | 0.1752 to 0.6381 |  | 0.0005 |
|  | Control vs. GAS/200 | 0.4733 |  | 0.2419 to 0.7048 |  | 0.0001 |
|  | Control vs. Imipramine | 0.3667 |  | 0.1352 to 0.5981 |  | 0.0014 |
|  | Saline vs. GAS/25 | 0.2233 |  | -0.008095 to 0.4548 |  | 0.0618 |
|  | Saline vs. GAS/50 | 0.5700 |  | 0.3386 to 0.8014 |  | <0.0001 |
|  | Saline vs. GAS/100 | 0.7833 |  | 0.5519 to 1.015 |  | <0.0001 |
|  | Saline vs. GAS/200 | 0.8500 |  | 0.6186 to 1.081 |  | <0.0001 |
|  | Saline vs. Imipramine | 0.7433 |  | 0.5119 to 0.9748 |  | <0.0001 |
|  | GAS/25 vs. GAS/50 | 0.3467 |  | 0.1152 to 0.5781 |  | 0.0023 |
|  | GAS/25 vs. GAS/100 | 0.5600 |  | 0.3286 to 0.7914 |  | <0.0001 |
|  | GAS/25 vs. GAS/200 | 0.6267 |  | 0.3952 to 0.8581 |  | <0.0001 |
|  | GAS/25 vs. Imipramine | 0.5200 |  | 0.2886 to 0.7514 |  | <0.0001 |
|  | GAS/50 vs. GAS/100 | 0.2133 |  | -0.01810 to 0.4448 |  | 0.0799 |
|  | GAS/50 vs. GAS/200 | 0.2800 |  | 0.04857 to 0.5114 |  | 0.0137 |
|  | GAS/50 vs. Imipramine | 0.1733 |  | -0.05810 to 0.4048 |  | 0.2112 |
|  | GAS/100 vs. GAS/200 | 0.06667 |  | -0.1648 to 0.2981 |  | 0.9496 |
|  | GAS/100 vs. Imipramine | -0.04000 |  | -0.2714 to 0.1914 |  | 0.9961 |
|  | GAS/200 vs. Imipramine | -0.1067 |  | -0.3381 to 0.1248 |  | 0.6996 |
| *Figure 6H* | Control vs. Saline | 0.7543 | One-Way ANOVA  Tukey’s post hoc tests | 0.4978 to 1.011 | F (6, 14) = 0.5416 | <0.0001 |
|  | Control vs. GAS/25 | 0.4010 |  | 0.1445 to 0.6574 |  | 0.0016 |
|  | Control vs. GAS/50 | -0.03003 |  | -0.2865 to 0.2264 |  | 0.9996 |
|  | Control vs. GAS/100 | -0.1993 |  | -0.4557 to 0.05720 |  | 0.1820 |
|  | Control vs. GAS/200 | -0.3202 |  | -0.5767 to -0.06376 |  | 0.0107 |
|  | Control vs. Imipramine | 0.4228 |  | 0.1664 to 0.6793 |  | 0.0009 |
|  | Saline vs. GAS/25 | -0.3533 |  | -0.6098 to -0.09686 |  | 0.0048 |
|  | Saline vs. GAS/50 | -0.7843 |  | -1.041 to -0.5279 |  | <0.0001 |
|  | Saline vs. GAS/100 | -0.9536 |  | -1.210 to -0.6971 |  | <0.0001 |
|  | Saline vs. GAS/200 | -1.075 |  | -1.331 to -0.8181 |  | <0.0001 |
|  | Saline vs. Imipramine | -0.3315 |  | -0.5879 to -0.07499 |  | 0.0082 |
|  | GAS/25 vs. GAS/50 | -0.4310 |  | -0.6875 to -0.1745 |  | 0.0008 |
|  | GAS/25 vs. GAS/100 | -0.6002 |  | -0.8567 to -0.3438 |  | <0.0001 |
|  | GAS/25 vs. GAS/200 | -0.7212 |  | -0.9777 to -0.4647 |  | <0.0001 |
|  | GAS/25 vs. Imipramine | 0.02187 |  | -0.2346 to 0.2783 |  | >0.9999 |
|  | GAS/50 vs. GAS/100 | -0.1692 |  | -0.4257 to 0.08723 |  | 0.3290 |
|  | GAS/50 vs. GAS/200 | -0.2902 |  | -0.5467 to -0.03373 |  | 0.0223 |
|  | GAS/50 vs. Imipramine | 0.4529 |  | 0.1964 to 0.7093 |  | 0.0005 |
|  | GAS/100 vs. GAS/200 | -0.1210 |  | -0.3774 to 0.1355 |  | 0.6787 |
|  | GAS/100 vs. Imipramine | 0.6221 |  | 0.3656 to 0.8786 |  | <0.0001 |
|  | GAS/200 vs. Imipramine | 0.7431 |  | 0.4866 to 0.9995 |  | <0.0001 |
| *Figure 6J* | Control vs. Saline | 0.8000 | One-Way ANOVA  Tukey’s post hoc tests | 0.6048 to 0.9952 | F (6, 14) = 0.5619 | <0.0001 |
|  | Control vs. GAS/25 | 0.2400 |  | 0.04484 to 0.4352 |  | 0.0121 |
|  | Control vs. GAS/50 | -0.02000 |  | -0.2152 to 0.1752 |  | 0.9998 |
|  | Control vs. GAS/100 | -0.2467 |  | -0.4418 to -0.05151 |  | 0.0098 |
|  | Control vs. GAS/200 | -0.1333 |  | -0.3285 to 0.06183 |  | 0.2944 |
|  | Control vs. Imipramine | 0.2100 |  | 0.01484 to 0.4052 |  | 0.0314 |
|  | Saline vs. GAS/25 | -0.5600 |  | -0.7552 to -0.3648 |  | <0.0001 |
|  | Saline vs. GAS/50 | -0.8200 |  | -1.015 to -0.6248 |  | <0.0001 |
|  | Saline vs. GAS/100 | -1.047 |  | -1.242 to -0.8515 |  | <0.0001 |
|  | Saline vs. GAS/200 | -0.9333 |  | -1.128 to -0.7382 |  | <0.0001 |
|  | Saline vs. Imipramine | -0.5900 |  | -0.7852 to -0.3948 |  | <0.0001 |
|  | GAS/25 vs. GAS/50 | -0.2600 |  | -0.4552 to -0.06484 |  | 0.0064 |
|  | GAS/25 vs. GAS/100 | -0.4867 |  | -0.6818 to -0.2915 |  | <0.0001 |
|  | GAS/25 vs. GAS/200 | -0.3733 |  | -0.5685 to -0.1782 |  | 0.0002 |
|  | GAS/25 vs. Imipramine | -0.03000 |  | -0.2252 to 0.1652 |  | 0.9980 |
|  | GAS/50 vs. GAS/100 | -0.2267 |  | -0.4218 to -0.03151 |  | 0.0185 |
|  | GAS/50 vs. GAS/200 | -0.1133 |  | -0.3085 to 0.08183 |  | 0.4646 |
|  | GAS/50 vs. Imipramine | 0.2300 |  | 0.03484 to 0.4252 |  | 0.0166 |
|  | GAS/100 vs. GAS/200 | 0.1133 |  | -0.08183 to 0.3085 |  | 0.4646 |
|  | GAS/100 vs. Imipramine | 0.4567 |  | 0.2615 to 0.6518 |  | <0.0001 |
|  | GAS/200 vs. Imipramine | 0.3433 |  | 0.1482 to 0.5385 |  | 0.0005 |
| *Figure 6K* | Control vs. Saline | 0.8767 | One-Way ANOVA  Tukey’s post hoc tests | 0.7466 to 1.007 | F (6, 14) = 0.1879 | <0.0001 |
|  | Control vs. GAS/25 | 0.1867 |  | 0.05661 to 0.3167 |  | 0.0034 |
|  | Control vs. GAS/50 | -0.006667 |  | -0.1367 to 0.1234 |  | >0.9999 |
|  | Control vs. GAS/100 | -0.2467 |  | -0.3767 to -0.1166 |  | 0.0002 |
|  | Control vs. GAS/200 | -0.1833 |  | -0.3134 to -0.05327 |  | 0.0040 |
|  | Control vs. Imipramine | 0.1900 |  | 0.05994 to 0.3201 |  | 0.0029 |
|  | Saline vs. GAS/25 | -0.6900 |  | -0.8201 to -0.5599 |  | <0.0001 |
|  | Saline vs. GAS/50 | -0.8833 |  | -1.013 to -0.7533 |  | <0.0001 |
|  | Saline vs. GAS/100 | -1.123 |  | -1.253 to -0.9933 |  | <0.0001 |
|  | Saline vs. GAS/200 | -1.060 |  | -1.190 to -0.9299 |  | <0.0001 |
|  | Saline vs. Imipramine | -0.6867 |  | -0.8167 to -0.5566 |  | <0.0001 |
|  | GAS/25 vs. GAS/50 | -0.1933 |  | -0.3234 to -0.06327 |  | 0.0025 |
|  | GAS/25 vs. GAS/100 | -0.4333 |  | -0.5634 to -0.3033 |  | <0.0001 |
|  | GAS/25 vs. GAS/200 | -0.3700 |  | -0.5001 to -0.2399 |  | <0.0001 |
|  | GAS/25 vs. Imipramine | 0.003333 |  | -0.1267 to 0.1334 |  | >0.9999 |
|  | GAS/50 vs. GAS/100 | -0.2400 |  | -0.3701 to -0.1099 |  | 0.0003 |
|  | GAS/50 vs. GAS/200 | -0.1767 |  | -0.3067 to -0.04661 |  | 0.0054 |
|  | GAS/50 vs. Imipramine | 0.1967 |  | 0.06661 to 0.3267 |  | 0.0021 |
|  | GAS/100 vs. GAS/200 | 0.06333 |  | -0.06673 to 0.1934 |  | 0.6485 |
|  | GAS/100 vs. Imipramine | 0.4367 |  | 0.3066 to 0.5667 |  | <0.0001 |
|  | GAS/200 vs. Imipramine | 0.3733 |  | 0.2433 to 0.5034 |  | <0.0001 |

**Supplementary Table 7. The F value and P value in multiple comparisons of figure 7**

| **Marker** | **Groups** | **Mean Diff.** | **Type of ANOVA** | **95.00% CI of diff.** | **F, DFn, Dfd** | **P value** |
| --- | --- | --- | --- | --- | --- | --- |
| *Figure 7C* | Control vs. Saline | 0.7767 | One-Way ANOVA  Tukey’s post hoc tests | 0.6034 to 0.9500 | F (4, 10) = 0.3497 | <0.0001 |
|  | Control vs. GAS | -0.1767 |  | -0.3500 to -0.003350 |  | 0.0453 |
|  | Control vs. ICG-001 | 0.3600 |  | 0.1867 to 0.5333 |  | 0.0003 |
|  | Control vs. IWP-2 | 0.6333 |  | 0.4600 to 0.8066 |  | <0.0001 |
|  | Saline vs. GAS | -0.9533 |  | -1.127 to -0.7800 |  | <0.0001 |
|  | Saline vs. ICG-001 | -0.4167 |  | -0.5900 to -0.2434 |  | <0.0001 |
|  | Saline vs. IWP-2 | -0.1433 |  | -0.3166 to 0.02998 |  | 0.1198 |
|  | GAS vs. ICG-001 | 0.5367 |  | 0.3634 to 0.7100 |  | <0.0001 |
|  | GAS vs. IWP-2 | 0.8100 |  | 0.6367 to 0.9833 |  | <0.0001 |
|  | ICG-001 vs. IWP-2 | 0.2733 |  | 0.1000 to 0.4466 |  | 0.0029 |
| *Figure 7D* | Control vs. Saline | 0.5033 | One-Way ANOVA  Tukey’s post hoc tests | 0.3941 to 0.6126 | F (4, 10) = 0.5032 | <0.0001 |
|  | Control vs. GAS | -0.2567 |  | -0.3659 to -0.1474 |  | 0.0001 |
|  | Control vs. ICG-001 | 0.1700 |  | 0.06074 to 0.2793 |  | 0.0032 |
|  | Control vs. IWP-2 | 0.3433 |  | 0.2341 to 0.4526 |  | <0.0001 |
|  | Saline vs. GAS | -0.7600 |  | -0.8693 to -0.6507 |  | <0.0001 |
|  | Saline vs. ICG-001 | -0.3333 |  | -0.4426 to -0.2241 |  | <0.0001 |
|  | Saline vs. IWP-2 | -0.1600 |  | -0.2693 to -0.05074 |  | 0.0049 |
|  | GAS vs. ICG-001 | 0.4267 |  | 0.3174 to 0.5359 |  | <0.0001 |
|  | GAS vs. IWP-2 | 0.6000 |  | 0.4907 to 0.7093 |  | <0.0001 |
|  | ICG-001 vs. IWP-2 | 0.1733 |  | 0.06407 to 0.2826 |  | 0.0028 |
| *Figure 7E* | Control vs. Saline | -0.4333 | One-Way ANOVA  Tukey’s post hoc tests | -0.8104 to -0.05624 | F (4, 10) = 0.2675 | 0.0234 |
|  | Control vs. GAS | 0.2600 |  | -0.1171 to 0.6371 |  | 0.2313 |
|  | Control vs. ICG-001 | -0.4567 |  | -0.8338 to -0.07957 |  | 0.0171 |
|  | Control vs. IWP-2 | -0.4500 |  | -0.8271 to -0.07290 |  | 0.0187 |
|  | Saline vs. GAS | 0.6933 |  | 0.3162 to 1.070 |  | 0.0009 |
|  | Saline vs. ICG-001 | -0.02333 |  | -0.4004 to 0.3538 |  | 0.9995 |
|  | Saline vs. IWP-2 | -0.01667 |  | -0.3938 to 0.3604 |  | 0.9999 |
|  | GAS vs. ICG-001 | -0.7167 |  | -1.094 to -0.3396 |  | 0.0007 |
|  | GAS vs. IWP-2 | -0.7100 |  | -1.087 to -0.3329 |  | 0.0008 |
|  | ICG-001 vs. IWP-2 | 0.006667 |  | -0.3704 to 0.3838 |  | >0.9999 |
| *Figure 7F* | Control vs. Saline | 0.4467 | One-Way ANOVA  Tukey’s post hoc tests | 0.2739 to 0.6194 | F (4, 10) = 0.2884 | <0.0001 |
|  | Control vs. GAS | -0.2767 |  | -0.4494 to -0.1039 |  | 0.0026 |
|  | Control vs. ICG-001 | 0.3033 |  | 0.1306 to 0.4761 |  | 0.0013 |
|  | Control vs. IWP-2 | 0.3533 |  | 0.1806 to 0.5261 |  | 0.0004 |
|  | Saline vs. GAS | -0.7233 |  | -0.8961 to -0.5506 |  | <0.0001 |
|  | Saline vs. ICG-001 | -0.1433 |  | -0.3161 to 0.02943 |  | 0.1182 |
|  | Saline vs. IWP-2 | -0.09333 |  | -0.2661 to 0.07943 |  | 0.4349 |
|  | GAS vs. ICG-001 | 0.5800 |  | 0.4072 to 0.7528 |  | <0.0001 |
|  | GAS vs. IWP-2 | 0.6300 |  | 0.4572 to 0.8028 |  | <0.0001 |
|  | ICG-001 vs. IWP-2 | 0.05000 |  | -0.1228 to 0.2228 |  | 0.8698 |
| *Figure 7G* | Control vs. Saline | 0.7867 | One-Way ANOVA  Tukey’s post hoc tests | 0.2742 to 1.299 | F (4, 10) = 0.3493 | 0.0035 |
|  | Control vs. GAS | -0.5967 |  | -1.109 to -0.08422 |  | 0.0216 |
|  | Control vs. ICG-001 | 0.4833 |  | -0.02911 to 0.9958 |  | 0.0668 |
|  | Control vs. IWP-2 | 0.5867 |  | 0.07422 to 1.099 |  | 0.0239 |
|  | Saline vs. GAS | -1.383 |  | -1.896 to -0.8709 |  | <0.0001 |
|  | Saline vs. ICG-001 | -0.3033 |  | -0.8158 to 0.2091 |  | 0.3542 |
|  | Saline vs. IWP-2 | -0.2000 |  | -0.7124 to 0.3124 |  | 0.7059 |
|  | GAS vs. ICG-001 | 1.080 |  | 0.5676 to 1.592 |  | 0.0003 |
|  | GAS vs. IWP-2 | 1.183 |  | 0.6709 to 1.696 |  | 0.0001 |
|  | ICG-001 vs. IWP-2 | 0.1033 |  | -0.4091 to 0.6158 |  | 0.9599 |
| *Figure 7H* | Control vs. Saline | 0.6233 | One-Way ANOVA  Tukey’s post hoc tests | 0.5430 to 0.7036 | F (4, 10) = 0.2829 | <0.0001 |
|  | Control vs. GAS | -0.3100 |  | -0.3903 to -0.2297 |  | <0.0001 |
|  | Control vs. ICG-001 | 0.3900 |  | 0.3097 to 0.4703 |  | <0.0001 |
|  | Control vs. IWP-2 | 0.5000 |  | 0.4197 to 0.5803 |  | <0.0001 |
|  | Saline vs. GAS | -0.9333 |  | -1.014 to -0.8530 |  | <0.0001 |
|  | Saline vs. ICG-001 | -0.2333 |  | -0.3136 to -0.1530 |  | <0.0001 |
|  | Saline vs. IWP-2 | -0.1233 |  | -0.2036 to -0.04302 |  | 0.0035 |
|  | GAS vs. ICG-001 | 0.7000 |  | 0.6197 to 0.7803 |  | <0.0001 |
|  | GAS vs. IWP-2 | 0.8100 |  | 0.7297 to 0.8903 |  | <0.0001 |
|  | ICG-001 vs. IWP-2 | 0.1100 |  | 0.02968 to 0.1903 |  | 0.0078 |
| *Figure 7K* | Control vs. Stress | 12.97 | One-Way ANOVA  Tukey’s post hoc tests | 4.589 to 21.35 | F (4, 20) = 0.2068 | 0.0014 |
|  | Control vs. GAS | -0.6992 |  | -9.081 to 7.683 |  | 0.9991 |
|  | Control vs. ICG-001 | 8.723 |  | 0.3409 to 17.10 |  | 0.0389 |
|  | Control vs. IWP-2 | 9.425 |  | 1.043 to 17.81 |  | 0.0229 |
|  | Stress vs. GAS | -13.67 |  | -22.05 to -5.289 |  | 0.0008 |
|  | Stress vs. ICG-001 | -4.248 |  | -12.63 to 4.133 |  | 0.5641 |
|  | Stress vs. IWP-2 | -3.547 |  | -11.93 to 4.835 |  | 0.7139 |
|  | GAS vs. ICG-001 | 9.422 |  | 1.040 to 17.80 |  | 0.0229 |
|  | GAS vs. IWP-2 | 10.12 |  | 1.742 to 18.51 |  | 0.0133 |
|  | ICG-001 vs. IWP-2 | 0.7018 |  | -7.680 to 9.084 |  | 0.9991 |
| *Figure 7L* | Control vs. Saline | 18.70 | One-Way ANOVA  Tukey’s post hoc tests | 8.997 to 28.40 | F (4, 20) = 0.3716 | 0.0001 |
|  | Control vs. GAS | 2.026 |  | -7.674 to 11.73 |  | 0.9693 |
|  | Control vs. ICG-001 | 13.45 |  | 3.748 to 23.15 |  | 0.0040 |
|  | Control vs. IWP-2 | 16.15 |  | 6.450 to 25.85 |  | 0.0006 |
|  | Saline vs. GAS | -16.67 |  | -26.37 to -6.971 |  | 0.0004 |
|  | Saline vs. ICG-001 | -5.248 |  | -14.95 to 4.451 |  | 0.5029 |
|  | Saline vs. IWP-2 | -2.547 |  | -12.25 to 7.153 |  | 0.9318 |
|  | GAS vs. ICG-001 | 11.42 |  | 1.722 to 21.12 |  | 0.0162 |
|  | GAS vs. IWP-2 | 14.12 |  | 4.424 to 23.82 |  | 0.0025 |
|  | ICG-001 vs. IWP-2 | 2.702 |  | -6.998 to 12.40 |  | 0.9170 |
| *Figure 7M* | Control vs. Saline | 5.737 | One-Way ANOVA  Tukey’s post hoc tests | 1.513 to 9.960 | F (4, 20) = 0.1117 | 0.0049 |
|  | Control vs. GAS | -1.386 |  | -5.610 to 2.838 |  | 0.8602 |
|  | Control vs. ICG-001 | 6.392 |  | 2.168 to 10.62 |  | 0.0017 |
|  | Control vs. IWP-2 | 4.116 |  | -0.1080 to 8.340 |  | 0.0584 |
|  | Saline vs. GAS | -7.123 |  | -11.35 to -2.899 |  | 0.0005 |
|  | Saline vs. ICG-001 | 0.6554 |  | -3.568 to 4.879 |  | 0.9897 |
|  | Saline vs. IWP-2 | -1.621 |  | -5.845 to 2.603 |  | 0.7794 |
|  | GAS vs. ICG-001 | 7.778 |  | 3.554 to 12.00 |  | 0.0002 |
|  | GAS vs. IWP-2 | 5.502 |  | 1.278 to 9.726 |  | 0.0071 |
|  | ICG-001 vs. IWP-2 | -2.276 |  | -6.500 to 1.948 |  | 0.5068 |
| *Figure 7N* | Control vs. Saline | 5.312 | One-Way ANOVA  Tukey’s post hoc tests | 0.9438 to 9.679 | F (4, 20) = 0.9396 | 0.0126 |
|  | Control vs. GAS | -1.386 |  | -5.754 to 2.982 |  | 0.8739 |
|  | Control vs. ICG-001 | 6.392 |  | 2.024 to 10.76 |  | 0.0024 |
|  | Control vs. IWP-2 | 4.116 |  | -0.2520 to 8.484 |  | 0.0708 |
|  | Saline vs. GAS | -6.698 |  | -11.07 to -2.330 |  | 0.0015 |
|  | Saline vs. ICG-001 | 1.080 |  | -3.287 to 5.448 |  | 0.9443 |
|  | Saline vs. IWP-2 | -1.196 |  | -5.564 to 3.172 |  | 0.9216 |
|  | GAS vs. ICG-001 | 7.778 |  | 3.410 to 12.15 |  | 0.0003 |
|  | GAS vs. IWP-2 | 5.502 |  | 1.134 to 9.870 |  | 0.0094 |
|  | ICG-001 vs. IWP-2 | -2.276 |  | 0.9438 to 9.679 |  | 0.5384 |
| *Figure 7O* | Control vs. Saline | 21.01 | One-Way ANOVA  Tukey’s post hoc tests | 5.958 to 36.07 | F (4, 20) = 0.6955 | 0.0026 |
|  | Control vs. GAS | -2.079 |  | -17.13 to 12.98 |  | 0.9945 |
|  | Control vs. ICG-001 | 15.53 |  | 0.4709 to 30.58 |  | 0.0405 |
|  | Control vs. IWP-2 | 18.17 |  | 3.110 to 33.22 |  | 0.0115 |
|  | Saline vs. GAS | -23.09 |  | -38.15 to -8.037 |  | 0.0008 |
|  | Saline vs. ICG-001 | -5.488 |  | -20.54 to 9.568 |  | 0.8312 |
|  | Saline vs. IWP-2 | -2.848 |  | -17.90 to 12.21 |  | 0.9820 |
|  | GAS vs. ICG-001 | 17.60 |  | 2.550 to 32.66 |  | 0.0152 |
|  | GAS vs. IWP-2 | 20.24 |  | 5.189 to 35.30 |  | 0.0039 |
|  | ICG-001 vs. IWP-2 | 2.639 |  | -12.42 to 17.69 |  | 0.9864 |
| *Figure 7P* | Control vs. Saline | 15.47 | One-Way ANOVA  Tukey’s post hoc tests | 3.005 to 27.93 | F (4, 35) = 0.8916 | 0.0088 |
|  | Control vs. GAS | -0.3606 |  | -12.82 to 12.10 |  | >0.9999 |
|  | Control vs. ICG-001 | 10.40 |  | -2.064 to 22.86 |  | 0.1395 |
|  | Control vs. IWP-2 | 9.659 |  | -2.802 to 22.12 |  | 0.1932 |
|  | Saline vs. GAS | -15.83 |  | -28.29 to -3.366 |  | 0.0071 |
|  | Saline vs. ICG-001 | -5.070 |  | -17.53 to 7.391 |  | 0.7682 |
|  | Saline vs. IWP-2 | -5.808 |  | -18.27 to 6.653 |  | 0.6687 |
|  | GAS vs. ICG-001 | 10.76 |  | -1.703 to 23.22 |  | 0.1180 |
|  | GAS vs. IWP-2 | 10.02 |  | -2.442 to 22.48 |  | 0.1653 |
|  | ICG-001 vs. IWP-2 | -0.7383 |  | -13.20 to 11.72 |  | 0.9998 |
| *Figure 7Q* | Control vs. Saline | 18.19 | One-Way ANOVA  Tukey’s post hoc tests | 4.179 to 32.20 | F (4, 35) = 0.2583 | 0.0057 |
|  | Control vs. GAS | 2.365 |  | -11.64 to 16.37 |  | 0.9882 |
|  | Control vs. ICG-001 | 25.21 |  | 11.20 to 39.22 |  | <0.0001 |
|  | Control vs. IWP-2 | 15.82 |  | -29.83 to -1.814 |  | 0.0005 |
|  | Saline vs. GAS | 22.52 |  | 8.515 to 36.53 |  | 0.0203 |
|  | Saline vs. ICG-001 | 7.021 |  | -6.988 to 21.03 |  | 0.6062 |
|  | Saline vs. IWP-2 | 4.336 |  | -9.673 to 18.34 |  | 0.8987 |
|  | GAS vs. ICG-001 | 22.84 |  | 8.835 to 36.85 |  | 0.0004 |
|  | GAS vs. IWP-2 | 20.16 |  | 6.150 to 34.17 |  | 0.0018 |
|  | ICG-001 vs. IWP-2 | -2.685 |  | -16.69 to 11.32 |  | 0.9811 |
| *Figure 7* | Control vs. Saline | -66.25 | One-Way ANOVA  Tukey’s post hoc tests | -114.2 to -18.28 | F (4, 35)  = 0.2219 | 0.0006 |
|  | Control vs. GAS | 12.75 |  | -35.22 to 60.72 |  | 0.9391 |
|  | Control vs. ICG-001 | -62.05 |  | -110.0 to -14.08 |  | 0.0059 |
|  | Control vs. IWP-2 | -60.01 |  | -108.0 to -12.04 |  | 0.0082 |
|  | Saline vs. GAS | 79.00 |  | 31.03 to 127.0 |  | 0.0003 |
|  | Saline vs. ICG-001 | 4.196 |  | -43.78 to 52.17 |  | 0.9991 |
|  | Saline vs. IWP-2 | 6.236 |  | -41.74 to 54.21 |  | 0.9957 |
|  | GAS vs. ICG-001 | -74.80 |  | -122.8 to -26.83 |  | 0.0007 |
|  | GAS vs. IWP-2 | -72.76 |  | -120.7 to -24.79 |  | 0.0010 |
|  | ICG-001 vs. IWP-2 | 2.040 |  | -114.2 to -18.28 |  | >0.9999 |
